# Innovations at the intersection of homelessness and substance use during the COVID-19 pandemic: a scoping review

**DOI:** 10.1186/s12954-025-01235-7

**Published:** 2025-07-29

**Authors:** Hannah Passmore, Sam Craft, Rachel Krieger, Sunny Tang, Sofia Sacerdote, Emily Lumbis, Stephanie Blaufarb, Kelly M. Doran

**Affiliations:** 1 Department of Emergency Medicine, NYU Grossman School of Medicine, 227 E 30 th Street, New York, NY 10016 USA; 2 Department of Population Health, NYU Grossman School of Medicine, 227 E 30 th Street, New York, NY 10016 USA

**Keywords:** Homelessness, Substance use, COVID-19, Harm reduction, Telemedicine, Overdose, Drug use

## Abstract

**Background:**

The COVID-19 pandemic led to disruptions in substance use and harm reduction services for people experiencing homelessness (PEH) as well as opportunities to innovate. Pandemic-era innovations may offer insights on more effective approaches to the intertwined issues of homelessness and substance use beyond the pandemic. We present findings from a scoping literature review of articles describing interventions related to substance use and homelessness that emerged during the pandemic.

**Methods:**

We conducted a scoping literature review to identify articles on pandemic-era innovations related to substance use and homelessness. We completed a comprehensive search for articles in nine academic and grey literature databases in November 2022, and a second database search in September 2023. We screened titles, abstracts, and full text using predefined inclusion and exclusion criteria. We extracted data on study design, location, participants, and outcomes.

**Results:**

Database searches yielded 812 unique articles; 68 met inclusion criteria. Most articles discussed interventions addressing opioid use (n = 60). Commonly described interventions included telemedicine-based prescribing of medications for opioid use disorder (MOUD), homeless services site-based MOUD provision, managed alcohol programs, supervised consumption services, and safer supply prescribing. Articles reported few intervention-related adverse effects, though study designs (e.g., non-experimental, observational studies lacking comparison groups) presented limitations to effectiveness outcome assessment. Surmountable challenges associated with interventions included inequitable access to technology for PEH.

**Conclusions:**

Innovations in programs that provide substance use treatment and harm reduction services to PEH were observed during the COVID-19 pandemic. Further evidence is needed to determine which COVID-19 pandemic-related innovations were most impactful and how they should be prioritized and continued post-pandemic.

**Supplementary Information:**

The online version contains supplementary material available at 10.1186/s12954-025-01235-7.

## Introduction

Homelessness affects over 1.25 million people in the United States [1], an estimated 235,000 people in Canada [2], and at least 13,955 people in England and Wales [3] annually. The bidirectional relationship between homelessness and substance use is well documented [4, 5]. For example, substance use can be a risk factor for entry into homelessness [6–8] and homelessness itself can lead to new substance use [5, 9, 10], increases in substance use [11, 12], and more risky use behaviors [13–15]. Even before the COVID-19 pandemic, people experiencing homelessness (PEH) experienced well-documented barriers to accessing substance use disorder (SUD) treatment and harm reduction services, including stigma, competing priorities related to housing instability, and issues with health insurance and transportation [16–20]. Adding to the urgency of the issue, drug overdose is a leading cause of death among PEH [21–27]. The current drug toxicity crisis in the United States and Canada, characterized by the increase in synthetic opioids in the illicit market, has contributed to high rates of overdose deaths [28, 29]. The COVID-19 pandemic emerged on top of this backdrop of serious homelessness and overdose crises.

Existing barriers to SUD treatment and harm reduction services for PEH were exacerbated during the COVID-19 pandemic [30–32]. Experts predicted that harmful pandemic impacts related to substance use might be more severe for PEH, given the potentially compounding effects of interruptions to services, pandemic-related isolation and boredom, and changes in the drug supply [33–35]. Evidence suggests that during the COVID-19 pandemic, PEH experienced overdoses at higher rates than their housed counterparts [36, 37]. Simultaneously, increased isolation due to service closures and social distancing may have contributed to adverse mental health outcomes for PEH [38, 39]. Shelter-in-place policies and staff shortages due to illness or deployment to other sectors, among other factors, resulted in some restrictions to and closures of substance use treatment and harm reduction services, which potentially impacted PEH disproportionately [21, 40, 41].

While the COVID-19 pandemic led to challenges related to the provision of substance use and harm reduction services, it also served as an impetus to creative innovations to adapt substance use care into new pandemic-appropriate models, including ones that centered the needs of PEH. Investigating these innovations can provide insight into new, potentially effective approaches for addressing the intertwined issues of homelessness and substance use in emergency situations and beyond. While other scoping and rapid reviews have investigated COVID-19 pandemic-related innovations related to SUD treatment and harm reduction [42, 43] and homeless services [44], to date, no review has specifically examined innovations at the *intersection* of homelessness and substance use. To fill this gap, we conducted a scoping review of interventions at this intersection, including programs providing SUD treatment or harm reduction services for PEH, that were newly created, expanded, or significantly adapted during the COVID-19 pandemic. In this review, we aimed to identify and summarize the literature on these pandemic innovations, with the goal of providing actionable insight for future pandemics and for improving substance use-related services for PEH more broadly.

## Methods

We completed a comprehensive survey of the available literature to capture the full scope of innovations taking place related to substance use (including treatment, overdose prevention and harm reduction, and other substance use-related interventions) for PEH during the COVID-19 pandemic. We followed the Levac et al. framework for scoping review methodology, which allowed us to incorporate a variety of article types to achieve our goal of broadly cataloging COVID-19 pandemic-era innovations [45]. We opted to conduct a scoping review rather than a systematic review because we wanted to be able to flexibly examine and summarize the broad range of emergent innovations; these innovations had been described in articles using various methodologies and often not including formal outcome assessment, and thus the literature did not lend itself to a systematic review or formal synthesis of intervention effectiveness. The senior author (KMD) is an expert in the field of homelessness and substance use and provided guidance at all stages of the review, including through identifying relevant sources and verifying completeness of the search. We additionally consulted a Study Advisory Board (SAB) to provide input on the scoping review and help ensure we were not missing critical topics or programs. The SAB included individuals with lived experiences of homelessness and substance use, as well as representatives from homeless services organizations, healthcare organizations, governmental agencies, and local and national advocacy organizations.

### Search strategy and study selection

We conducted a search of five databases (CINAHL, PubMed, PsycINFO, EMBASE, and Web of Science) for articles on COVID-19 pandemic-era innovations—including new programs and modifications to existing programs—at the intersection of substance use and homelessness. The full search strategy (including search terms), which was developed with the assistance of a research librarian, is provided in Appendix A. The results of the search were uploaded into the literature review management tool Covidence [46], which was used to facilitate the article organization, screening, and data extraction processes.

We conducted the initial search in November 2022. To ensure inclusion of recently published literature, we completed a second database search in September 2023 using the same process as the prior search.

We additionally completed a grey literature search in three grey literature databases (OAIster, New York Academy of Medicine, and OpenGrey) using the same search terms. We also conducted a tailored Google search for additional articles, using abridged search terms to comply with Google search limits (search terms are provided in Appendix A). To ensure the Google search results were not impacted by search optimization, we turned off search customization and cleared cookies before conducting the search. We reviewed the first 100 results for relevance. Finally, we reviewed the references of all included articles to identify any additional relevant articles not found via the other search strategies.

We included articles involving a new, modified, or expanded intervention (program or other initiative) addressing substance use (drug and/or alcohol use) among PEH during the COVID-19 pandemic. We did not restrict article inclusion to interventions that served only PEH; interventions could serve PEH along with other clients and there were not inclusion criteria for the number or proportion of participants who had to be PEH. However, in keeping with the goals of the review, articles not specifically focused on PEH had to contain some “meaningful discussion” of how interventions served PEH, which we defined broadly as any explicit discussion of how the needs of PEH were targeted or addressed. Articles were excluded if they were published before February 2020, only addressed tobacco or nicotine use (and not other drug use or alcohol use), did not describe any intervention or included only an intervention that did not directly address substance use (e.g., eviction moratoria, infection prevention measures), did not meaningfully discuss serving PEH, were published in a language other than English, or if the intervention was not in response to the COVID-19 pandemic (e.g., articles describing interventions that had started pre-pandemic but were published after February 2020). We excluded review articles that did not describe a unique intervention and articles published as abstracts only; there were no other exclusion criteria based on study design or article format (e.g., full research articles, brief reports).

Each article was first screened independently by two reviewers (RK, ST, HP, and/or SS) for relevance at the title and abstract level. Any conflicts were resolved via discussion, with the PI (KMD) adjudicating as needed. The full text for articles found to be relevant at the title and abstract level was screened independently by two reviewers (RK, ST, HP, and/or SS) for inclusion in the review; any conflicts were resolved using the aforementioned process.

### Data extraction and analysis

We extracted data from eligible articles using a tailored data extraction template on the Covidence platform. The data extraction template was developed by RK and revised in collaboration with the study team. Data extraction was completed by one study team member (RK, ST, HP, or SS) and checked for accuracy by an additional team member (RK, ST, HP, SS, EL, or KMD). Data extracted included intervention location, setting, purpose, methods, type of intervention, type of substance use addressed, participant demographics, key outcomes, and adverse effects. Given the varied methodologies and types of data reported in the included articles, we took a flexible approach to extracting relevant data present in each article without requiring data be reported using specific formats or units; absence of data or of certain types of data was also noted as appropriate for each article. The full data extraction template can be found in Appendix B.

We first conducted basic descriptive analyses of the extracted data to summarize characteristics (including methods, location, setting, type of substance use addressed, and type of intervention) of the included articles. We then used a textual synthesis approach [47] to summarize the main content of each article—including findings or descriptive information—in relation to the interventions discussed. We did not complete formal study quality assessments, in keeping with standard scoping review methodology and due to the wide range of design of included studies [48]. Instead, we provide details of each article’s methodology, including study design and sample size, and summarize articles’ limitations.

## Results

### Study selection

**Fig. 1:**
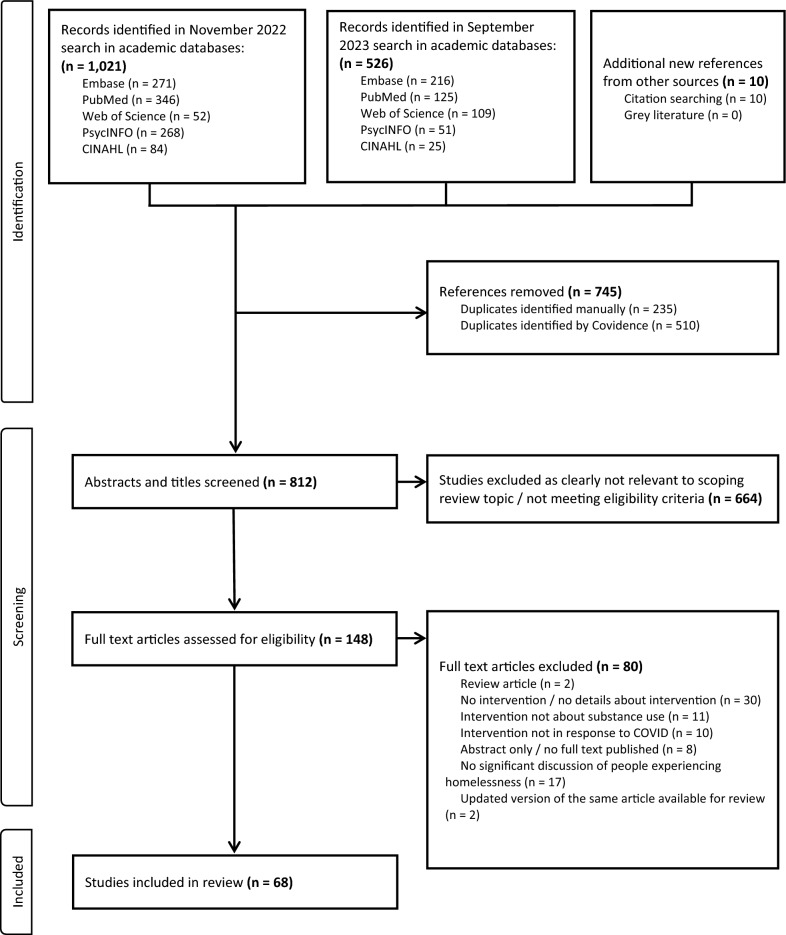
Preferred Reporting Items for Systematic Reviews and Meta-analyses extension for scoping review (PRISMA-ScR) flow diagram for study identification, screening, and inclusion

Figure 1 presents the PRISMA flowchart outlining the article identification, screening, and exclusion process. A total of 1557 articles were identified in database searches and through citation searching of the included articles. A total of 745 duplicate articles were identified and removed (510 identified automatically by the literature review software Covidence [46] and 235 identified via manual review by RK). We screened the titles and abstracts of the remaining 812 articles for eligibility. We excluded 664 irrelevant articles. We reviewed the full text of the remaining 148 articles. Following the full text review, we excluded an additional 80 articles. A full list of articles excluded following full text review, with reasons for exclusion, can be found in Appendix C. The remaining 68 articles were included in this scoping review. No additional articles meeting inclusion criteria were identified in the grey literature search or from consultation with experts.

### Article characteristics

The 68 articles included in the review described 86 relevant interventions. For the purposes of this review, we separated articles into the following methodological categories: program descriptions (i.e., articles that provided an overview of the intervention with or without basic descriptive data on clients/outcomes; n = 26), qualitative studies (n = 16), quantitative analytic studies (i.e., articles reporting results of formal quantitative analyses of participant characteristics or intervention outcomes; n = 14), case series/reports (n = 8), and mixed methods (i.e., articles combining both qualitative and quantitative analyses; n = 4). Most articles (n = 60) did not include a control or comparison group.

Characteristics of the included articles, including methods, year of publication, and types of substances addressed, are displayed in Table 1. Fourteen countries were represented; the countries with the most articles included in the review were the United States (n = 42) and Canada (n = 10). Most articles described interventions related to opioid use (n = 60). Most articles (n = 47) described interventions that were newly created during the COVID-19 pandemic; others (n = 21) described existing programs that substantially changed in response to the pandemic. Table 2 presents details of the articles, including intervention location, description of the intervention, substances addressed by the intervention, and number of participants described in the article.

**Table 1:** Characteristics of all included articles (n = 68)

	N (%)
Location	
North America	49 (72.1)
United States only	39 (79.6)
Canada only	7 (14.3)
United States and Canada	3 (6.1)
Europe	13 (19.1)
Africa	3 (4.4)
Australia	2 (2.9)
North America, Europe, and Asia	1 (1.5)
Setting	
Urban	57 (83.8)
Rural	1 (1.5)
Mixed/multiple (urban, suburban, rural)	10 (14.7)
Substance use type addressed	
Opioids only	38 (55.9)
Alcohol only	4 (5.9)
Opioids and alcohol only	11 (16.2)
Opioids and other drugs only	3 (4.4)
Opioids, other drugs, and alcohol	8 (11.8)
Not specified	4 (5.9)
Methods	
Program description^a^	26 (38.2)
Qualitative	16 (23.5)
Quantitative analytic study (cohort, case control, cross-sectional, etc.)	14 (20.6)
Case series and reports	8 (11.8)
Mixed methods	4 (5.9)
Specific to PEH^b^	34 (50.0)
Year published	
2020	5 (7.4)
2021	24 (35.3)
2022	26 (38.2)
2023	13 (19.1)

^a^Refers to articles that provide an overview of the intervention with or without basic summary statistics describing the characteristics of the clients/outcomes

^b^Refers to articles that described interventions designed specifically for and only serving PEH. Other interventions served PEH along with other clients

**Table 2:** List of all included articles (n = 68)

Author(s), year	Location	Type of intervention	Substance addressed by intervention	Number of participants^a^
Program description^b^ (n = 26)				
Dunn et al. 2022 [49]	London, England	Buprenorphine and methadone dispensing and harm reduction supply provision at protective hotel for PEH	Opioids	35
Fuertes et al. 2021 [50]	Lisbon, Portugal	Alcohol and OUD treatment, harm reduction services, and access to mobile drug consumption room for emergency shelter	Alcohol; Opioids; Stimulants	N/A
Harris, Young et al. 2021 [51]	Boston, USA and Toronto, Canada	Toronto: I&Q/respite unit with low-barrier MAP, prescribed safer supply, naloxone and syringe distribution, SCS, methadone and buprenorphine provision, and alcohol withdrawal management. Boston: I&Q/respite unit with naloxone and syringe distribution at discharge, onsite methadone administration, and alcohol withdrawal management	Alcohol; Opioids	N/A
Harris, Peterkin et al. 2021 [52]	Vancouver, Canada; Portland, Boston, and New Haven, USA	Vancouver: SUD management in I&Q units, longer buprenorphine (bupe) prescriptions, outpatient BZD tapers for alcohol withdrawal, safer supply prescribing, virtual supervision; Portland: telemedicine-based bupe prescribing, outpatient BZD tapers, pre-paid phone distribution; Boston: telemedicine-based bupe prescribing, outpatient BZD tapers, SUD management in I&Q units, pre-paid phone distribution; New Haven: telemedicine-based bupe prescribing, outpatient BZD tapers, SUD management in I&Q units	Alcohol; Opioids	N/A
Heimer et al. 2020 [53]	New Haven, Connecticut, USA	Buprenorphine and methadone delivery, methadone THDs, COVID-19 respite facility for PEH with harm reduction for alcohol and other substances, onsite methadone (via delivery)	Alcohol; Opioids; Stimulants	N/A
Kennedy et al. 2022 [54]	Los Angeles, California, USA	Low-threshold telephone addiction consultation service accessible via outreach and other providers, including medication (e.g. buprenorphine, naltrexone) prescribing	Alcohol; Opioids	557
Komaromy, Harris et al. 2021 [55]	Boston, Massachusetts, USA	Methadone and buprenorphine induction, maintenance, and dispensing, and SUD withdrawal management at hospital-based I&Q/respite site for PEH	Alcohol; Opioids; Stimulants; Benzodiazepines	226
Komaromy, Tomanovich et al. 2021 [56]	Boston, Massachusetts, USA	I&Q/respite unit with onsite SUD treatment, telemedicine-based SUD services, extended prescriptions and use of injectable buprenorphine, phone provision, street-based harm reduction outreach	Alcohol; Opioids; Stimulants; Benzodiazepines	N/A
Landgraf et al. 2020 [57]	Delaware, USA	Transitional housing hotel placement program for PEH enrolled or willing to enroll in SUD treatment	Not specified	332
Leo et al. 2021 [58]	Chicago, Illinois, USA	Mobile van-based buprenorphine induction	Opioids	N/A
Lynch et al. 2023^c^ [59]	Los Angeles, California, USA	Comprehensive co-located care at sanctioned encampment	Opioids	95
Malmusi et al. 2022 [60]	Barcelona, Spain	SUD services at emergency shelters	Alcohol; Opioids	N/A
Marcus et al. 2020^d^ [61]	Tshwane, South Africa	Methadone provision at an emergency temporary congregate shelter	Opioids	N/A
Messmer et al. 2023 [62]	Chicago, Illinois, USA	Mobile medical unit with low-threshold buprenorphine induction, maintenance, and dispensing	Opioids	587
O’Carroll et al. 2021 [63]	Dublin, Ireland	Methadone THDs, BZD maintenance treatment, naloxone provision at protective and I&Q hotels for PEH	Opioids; Benzodiazepines	N/A
Nash et al. 2022 [64]	Sydney, Australia	MAP, MOUD, and alcohol withdrawal management at I&Q site	Alcohol; Opioids	58
Quiñones et al. 2021 [65]	Puerto Rico, USA	Telemedicine-based buprenorphine induction and maintenance	Opioids	N/A
Ristau et al. 2021 [66]	San Francisco, California, USA	MAP at protective and I&Q hotels for PEH	Alcohol	26
Rottnek & Laxton, 2021 [67]	St Louis, Missouri, USA	Outreach-based MOUD delivery	Opioids	N/A
Sivakumar et al. 2022 [68]	New Haven, Connecticut, USA	HCV, HIV, and OUD co-treatment with telemedicine-based buprenorphine induction, linkage to methadone	Opioids	35
Tofighi et al. 2022 [69]	New York, New York, USA	Low-threshold telemedicine-based buprenorphine induction and maintenance	Opioids	78
Tringale & Subica, 2021 [70]	Los Angeles, California, USA	“Telephone booth” clinic-based telemedicine buprenorphine induction and maintenance, dispensing through coordinated pharmacy	Opioids	N/A
Venter & Heese, 2020 [71]	Tshwane, South Africa	Low-threshold methadone provision at emergency congregate shelter	Opioids	52
Wang, C.Y. et al. 2021 [72]	Chicago, Illinois, USA	Telemedicine-based buprenorphine induction and maintenance and methadone delivery at I&Q site for PEH	Opioids	51
Wang, L. et al. 2021 [73]	Ithaca and Manhattan, New York, USA	Low-threshold outreach and telemedicine-based buprenorphine induction and maintenance	Opioids	N/A
Yeo et al. 2021 [74]	Washington DC, USA	Low-threshold audio-only telemedicine-based buprenorphine induction and maintenance	Opioids	277
Qualitative (n = 16)				
Aronowitz et al. 2021 [75]	Philadelphia, Pennsylvania, USA	Telemedicine-based buprenorphine induction and maintenance, methadone THDs, street-based harm reduction outreach	Opioids	30
Brocious et al. 2021 [76]	Juneau, Alaska, USA	MAP at I&Q site for PEH	Alcohol	5
Darnton et al. 2023 [77]	Washington State, USA	Methadone THDs with video-observed dosing	Opioids	15
Harris et al. 2022 [78]	Boston, Massachusetts, USA	Methadone THDs and telemedicine-based buprenorphine induction and maintenance	Opioids	20
Kesten et al. 2021 [79]	Bristol, England	Telemedicine and outreach-based SUD treatment and harm reduction services, low-barrier buprenorphine and methadone provision, extended prescriptions, methadone THDs	Opioids	28
Lenhard et al. 2022 [80]	Cambridge, England	Telemedicine, relaxed alcohol consumption rules at protective hotels for PEH	Alcohol	N/A
Lockard et al. 2022 [81]	Portland, Oregon, USA	Low-threshold telemedicine-based buprenorphine induction and maintenance	Opioids	19
May et al. 2022 [82]	United Kingdom	Harm reduction supply delivery, methadone storage boxes, extended MOUD prescriptions, methadone THDs, telemedicine-based SUD assessment and services	Opioids	36
McCann et al. 2022 [83]	Kingston, Canada	Onsite SCS and harm reduction supply provision at emergency congregate shelter	Opioids	30
Neale et al. 2022 [84]	London, England	Buprenorphine and methadone provision and MAP at protective hotel for PEH	Alcohol; Opioids	34
Parkes et al. 2021 [85]	Edinburgh, Scotland	Harm reduction equipment delivery, methadone THDs, telemedicine-based support groups and appointments, naloxone distribution	Alcohol; Opioids	20
Parkes et al. 2021 [86]	Edinburgh, Scotland	Telemedicine-based support groups and appointments, phone provision, provision of safer injection equipment, naloxone provision, same-day drop-in SUD medication induction	Alcohol; Opioids	20
Russell et al. 2021 [87]	Canada	Expanded MOUD accessibility, telemedicine-based MOUD prescribing and SUD counseling	Opioids	196
Schofield et al. 2022 [88]	Scotland	Methadone THDs, expanded harm reduction equipment provision, shift to buprenorphine, telemedicine-based recovery support services	Opioids	29
Suen et al. 2022 [89]	San Francisco, California, USA	Expanded access to methadone THDs	Opioids	30
Wyatt et al. 2022 [90]	California, USA	Methadone THDs at protective hotel for PEH	Opioids	30
Quantitative analytic study (cohort, case control, cross-sectional, etc.) (n = 14)				
Azevedo et al. 2023 [91]	Lisbon, Portugal	Low-threshold pharmacological alcohol withdrawal management at emergency shelters	Alcohol	69
Fleming et al. 2022 [92]	San Francisco, California, USA	Buprenorphine induction at protective hotel for PEH	Opioids	686
Frost et al. 2022 [93]	USA	Telemedicine for buprenorphine	Opioids	17,182
Fuchs et al. 2021 [94]	San Francisco, California, USA	MOUD, MAP, and harm reduction services at I&Q hotel for PEH	Alcohol; Opioids; Marijuana; Stimulants	1009
Horig et al. 2023 [95]	Berlin, Germany	MOUD, MAP at specialized I&Q facility for PEH	Alcohol; Opioids	139
Huggett et al. 2021 [96]	Chicago, Illinois, USA	Onsite methadone (via delivery), buprenorphine and naltrexone induction and maintenance at protective hotel for PEH	Alcohol; Opioids	259
King et al. 2023 [97]	New York, New York, USA	Telephonic outreach program with peer support, counseling, referrals to medical providers	Alcohol; Opioids; Other	329
Lew et al. 2022 [98]	Hamilton, Canada	Safer use space, prescribed safer supply, MOUD, and harm reduction supplies at congregate shelter	Opioids	N/A
Matthews et al. 2023 [99]	New York, New York, USA	Telemedicine-based counseling for SUD	Not specified	370
Nordeck et al. 2021 [100]	Baltimore, Maryland, USA	Low-threshold telemedicine-based buprenorphine induction and maintenance and extended prescriptions	Opioids	322
Peterkin et al. 2023 [101]	Boston, Massachusetts, USA	Pre-paid cell phone provision for patients with SUD	Not specified	181
Siemens et al. 2023 [102]	Tshwane, South Africa	Low-threshold methadone provision at emergency congregate shelter	Opioids	495
Toseef et al. 2022 [103]	Denver, Colorado, USA	Telemedicine appointments (provider, therapist) for SUD	Not specified	1,626
Vorberg et al. 2023 [104]	Hamburg, Germany	Low-threshold clinic-based methadone provision	Opioids	84
Case series and reports (n = 8)				
Brothers et al. 2022 [105]	Halifax, Canada	Prescribed safer supply, MAP, buprenorphine and methadone at I&Q hotel for PEH	Alcohol; Opioids; Stimulants; Benzodiazepines	77
Flavin et al. 2022 [106]	New York, New York, USA	Low-threshold telemedicine-based buprenorphine treatment	Opioids	1
Harris et al. 2020 [107]	Boston, Massachusetts, USA	Low-threshold outreach and telemedicine-based buprenorphine induction and maintenance	Opioids	2
Hong et al. 2022 [108]	British Columbia, Canada	Safer supply prescribing at I&Q hotel for PEH	Opioids; Stimulants	1
Levander et al. 2022 [109]	Portland, Oregon, USA	Low-threshold telemedicine-based buprenorphine induction and maintenance	Opioids	3
Mehtani et al. 2021 [110]	San Francisco, California, USA	Telemedicine-based buprenorphine induction and maintenance at protective hotel for PEH	Opioids	12
Samuel et al. 2022 [111]	San Francisco, California, USA	Buprenorphine induction and delivery at protective hotel for PEH	Opioids	3
Scallan et al. 2022 [112]	Hamilton, Canada	Methadone provision (via delivery) at protective hotel for PEH	Opioids	2
Mixed methods (n = 4)				
Abbs et al. 2023 [113]	San Francisco, California, USA	MAP, buprenorphine delivery and harm reduction services at protective hotel for PEH	Alcohol; Opioids	346 (cross sectional analysis), 5 (focus group)
Bower et al. 2023 [114]	Sydney, Australia	Telemedicine-based SUD counseling	Alcohol; Opioids; Marijuana; Stimulants	64
Galarneau et al. 2023 [115]	Edmonton, Canada	Overdose prevention site at emergency congregate shelter	Opioids; Stimulants	45
Wiessing et al. 2022 [116]	Saskatchewan, Canada; Athens, Greece; Dublin, Ireland; Tel Aviv, Israel; Luxembourg, Bucharest; Romania, Glasgow, Scotland; Indiana, Minnesota, Ohio, Oregon, Pennsylvania, and Washington, USA	Methadone THDs, telemedicine and outreach-based SUD treatment, delivery of safe consumption supplies, emergency congregate shelters, protective hotels for PEH	Opioids	13 sites

^a^Number refers to study participants if available (e.g., for quantitative or qualitative studies) or intervention recipients if no specific number of study participants provided or applicable (e.g., for descriptions). Studies noted as N/A in this column did not provide singular information on the total number of intervention recipients (or provided estimates only) or had a study design for which the number was not applicable

^b^Refers to articles that provide an overview of the intervention with or without basic summary statistics describing the characteristics of the clients/outcomes

^c^This study provided a program description along with more detailed information about one recipient case

^d^This program description included information from 3 key informant interviews along with primary documentary data and internal memos

BZD benzodiazepine, I&Q isolation and quarantine, MAP managed alcohol program, MOUD medications for opioid use disorder, OUD opioid use disorder, PEH people experiencing homelessness, SCS supervised consumption services, SUD substance use disorder, THD take-home dose

The interventions described in the articles included in the review fit into two broad categories: (1) Interventions focused on SUD treatment, including medication and/or counseling based-treatment, and (2) Interventions focused on harm reduction, including preventing overdose and/or withdrawal from drugs and/or alcohol. Many of the interventions included elements from both categories. Table 3 summarizes key findings reported in the included articles by intervention type.

**Table 3:** Summary of key findings reported by articles included in the scoping review (n = 68)

Type of intervention (number of articles)	Summary of key findings
Substance use disorder treatment interventions	
Telemedicine-based MOUD induction and maintenance (n = 24)	• Majority of articles focused on buprenorphine [52, 54, 56, 65, 68–70, 72–75, 78, 81, 93, 100, 106, 107, 109, 110] • Telemedicine facilitated increased access and reduced barriers to MOUD [54, 81, 75, 100, 107, 110] • Technology access can be a barrier for PEH [52, 69, 75, 100]. This barrier can be mitigated by strategies including “facilitated” telemedicine [65, 70, 75, 107, 109], telephone provision [101], and audio-only visits [74, 100, 106] • Reported impacts on retention rates were largely positive [69, 100, 110] though one study suggested PEH may not receive equal benefits to people who are housed [103]
Telemedicine-based SUD counseling (n = 7)	• Telemedicine can increase engagement with SUD services for PEH [82] • Some articles noted retention challenges for PEH for telemedicine counseling [97, 99]
Mobile unit and street outreach-based MOUD induction and maintenance (n = 7)	• Articles noted successes of programs designed to literally “reach people where they are” (e.g., on the streets via street outreach) in connecting PEH to SUD treatment including MOUD [58, 62, 67, 73]
Buprenorphine and methadone provision in pandemic homeless services settings (n = 21)	• New models of onsite delivery and provision of MOUD were started during the pandemic in sites serving PEH (e.g., I&Q sites, protective hotel sites, shelters) [49, 51—53, 55, 56, 59, 61, 64, 71, 84, 92, 94–96, 98, 102, 105, 111–113] • Articles noted success in promoting treatment initiation [64, 95, 111, 113] and retention/adherence for those already taking buprenorphine or methadone [64, 96, 112]
Methadone take-home dosing and low-barrier provision (n = 14)	• Thirteen articles described expansion of take-home doses (TDHs) [53, 63, 75, 77–79, 82, 85, 87, 88–90, 116] and one article examined a low-barrier approach to methadone treatment [104] • Patients newly accessing take-home doses (THDs) reported feeling more control and autonomy [79, 82, 87, 90] • Articles noted potential destabilizing effect of THDs versus daily retrievals for some PEH [79, 82] • Some PEH were unable to access THDs due to their housing status [78]
Treatment for alcohol use disorder (n = 4)	• AUD treatment was offered at I&Q sites and protective hotels [51, 56, 96] and via telemedicine [54]
Benzodiazepine maintenance treatment (n = 1)	• One article discussed benzodiazepine maintenance for patients with benzodiazepine dependence; reported benefits were improved health and behavior, and facilitation of isolation/protective hotel stays [63]
Harm reduction interventions	
Managed alcohol programs (n = 9)	• MAPs provided set doses of alcohol to patients at I&Q sites [51, 94,95, 64, 66, 76, 105] and protective hotels [66, 113, 84] • Articles noted success of MAPs in preventing withdrawal and hospitalization [64, 76], with few reported adverse events 64, 66, 105]
Alcohol withdrawal management (n = 8)	• Articles most commonly discussed pharmacological alcohol withdrawal prevention and management (e.g., with benzodiazepines) in I&Q and protective sites serving PEH [50, 51, 55, 64, 91] • Articles reported acceptability and apparent successes of these programs in preventing severe withdrawal [64]
Supervised consumption services (n = 4)	• Articles described programs located in I&Q sites for PEH and shelters in Canada [51, 83, 98, 115] • Articles noted success in preventing overdose deaths [51, 115] without reported adverse events • Appeared to be widely used [83, 115]
Safer supply prescribing (n = 5)	• All articles described programs located in I&Q sites for PEH, shelters, or in hospital settings in Canada [51, 52, 98, 105, 108] • Programs appeared to be acceptable [105, 108], with few reported adverse events [105]

*AUD* alcohol use disorder, *I&Q* isolation and quarantine, *MAP* managed alcohol program, *MOUD* medications for opioid use disorder, *PEH* people experiencing homelessness, *SUD* substance use disorder, *THD* take-home dose

#### 1) Substance use disorder treatment interventions

In this section, we discuss articles describing interventions focused on SUD treatment that were created or substantially modified during the COVID-19 pandemic. These SUD treatment interventions included: (1a) telemedicine-based MOUD induction and maintenance (n = 24 articles); (1b) telemedicine-based SUD counseling (n = 7); (1c) mobile unit and street outreach-based MOUD induction and maintenance (n = 7); (1d) buprenorphine and methadone provision in pandemic homeless services settings (n = 21); (1e) methadone take-home dosing (THD) and low-barrier provision (n = 14); (1f) treatment for alcohol use disorder (AUD) (n = 4); and (1g) benzodiazepine maintenance treatment (n = 1).

##### 1a) Telemedicine-based MOUD induction and maintenance

The most common type of intervention discussed in the included articles was telemedicine-based MOUD induction and maintenance (n = 24) [52, 54, 56, 65, 68–70, 72–75, 78, 79, 81, 87, 93, 100, 101, 103, 106, 107, 109, 110, 116]. Most of these articles (n = 19) specifically discussed buprenorphine prescribing [52, 54, 56, 65, 68–70, 72–75, 78, 81, 93, 100, 106, 107, 109, 110]; the others discussed MOUD more broadly or did not specify the medications offered [79, 87, 101, 103, 116]. Articles reported several benefits of telemedicine-based buprenorphine prescribing, including increased access to and approachability of treatment [75, 107], and reduced barriers to care [81]. For example, in a qualitative study of 30 SUD treatment, harm reduction, and homelessness services providers and advocates in Philadelphia (USA), participants reported that telemedicine-based buprenorphine prescribing increased access, especially for patients who had difficulty traveling to appointments [75]. Another qualitative study that included interviews with 19 patients (21% PEH) receiving care at a low-threshold addiction treatment clinic in Portland, Oregon (USA) similarly found that telemedicine appointment availability was associated with improved access to care, as PEH could complete appointments in places such as encampments or cafés that were easier for them to access than a clinic [81].

Articles reported successes in prescribing buprenorphine to PEH via telemedicine. For example, a study in Baltimore (USA) reported 143 (21% PEH) successful telemedicine-based buprenorphine inductions between March and May 2020 [100]. An article describing a low-barrier telephone consultation program in Los Angeles (USA) reported that the program reached 557 patients and prescribed 662 medications (90% of which were buprenorphine) for SUD; about half of the patients served were PEH [54]. A case series of patients staying at COVID-19 Isolation & Quarantine (I&Q) sites for PEH in San Francisco (USA) discussed a novel “Telehealth Addiction Program” that provided consults and buprenorphine prescribing and maintenance via on-demand telemedicine visits; the program resulted in 12 patients (all of the patients who had OUD and were not already taking MOUD of 59 I&Q site guest consults) receiving new buprenorphine prescriptions via telemedicine [110]. In summary, multiple articles reported success in helping PEH start and continue treatment with buprenorphine via telemedicine.

On the other hand, articles also reported on some challenges faced by PEH related to telemedicine, including limited telephone access, lack of consistent telephone numbers, and other technological issues [52, 69, 75, 100]. One mitigation strategy described in a qualitative study with harm reduction, SUD treatment, and homeless services staff and providers in Philadelphia (USA) was “facilitated” telemedicine, in which patients could come into a clinic and use the clinic’s technology to be connected to a provider offsite [75]. Other articles described similar methods to mitigate technological barriers to telemedicine. For example, an article detailing a “telephone booth” model of facilitated telemedicine buprenorphine prescribing at a low-barrier clinic serving PEH in Los Angeles (USA) explained how patients visiting the clinic were provided with technology and assistance to connect via telemedicine to providers in a different part of the clinic building; the authors reported that the model was able to serve patients at a similar rate to pre-pandemic levels [70]. The authors of a case series in Boston (USA) similarly noted that facilitated telemedicine, in which harm reduction specialists met with patients and used their own devices to assist with video-conferencing with buprenorphine providers, allowed them to serve patients who would otherwise have difficulty accessing telemedicine [107]. In another study in Boston (USA), researchers found that providing SUD patients with pre-paid telephones was associated with significant increases in completed telemedicine visits [101]. Other studies reported success with buprenorphine prescribing using audio-only telephone calls, a lower-barrier option versus web/video conferencing for some PEH [106, 100, 74]. For example, a study (n = 277, 58% PEH or unstably housed) of the audio-only telephone buprenorphine prescribing program in Washington, DC (USA) reported that 111 patients were started on buprenorphine between March and December 2020, and 65% were retained in care as of December 2020 [74].

Less commonly noted challenges associated with telemedicine prescribing of MOUD included difficulties in building relationships with providers. A qualitative study with 28 individuals who used drugs in Bristol (England) (22 of whom were PEH or unstably housed) reported a concern that telemedicine was “impersonal” and made it difficult to form connections with providers [79]. Participants (n = 196, 25% PEH or unstably housed) of a national qualitative study of people who use drugs across Canada similarly reported that telemedicine-based prescribing was convenient but that it was harder to establish relationships with providers via telemedicine versus in person [87].

Articles that examined treatment retention for telemedicine-based buprenorphine prescribing programs reported largely positive results. In a Baltimore (USA) study, 64% of patients initiated on buprenorphine via telemedicine were retained in care after 30 days, which authors noted did not appear to differ from retention for patients enrolled in-person; 25% of patients in the study were PEH, and there were no significant retention differences by housing status [100]. An article describing a telemedicine-based buprenorphine clinic in New York City (USA) reported a 54% 2-month retention rate for patients initiated on buprenorphine via telemedicine; 72% of the patients served by the clinic were unstably housed [69]. Similarly, the study of the I&Q site “Telehealth Addiction Program” in San Francisco (USA) reported that 64% of patients prescribed buprenorphine via telemedicine remained in treatment at the time of discharge from the I&Q site [110]. On the other hand, a study in Washington, DC (USA) found lower retention rates for patients initiated on buprenorphine via telemedicine versus in-person (68% vs 94% retained at 90 days); given the study methods, it is unclear if this difference is due to the intervention modality or differences in patient baseline characteristics [74].

Importantly, a few articles suggested that PEH may not have experienced the same retention-related benefits of telemedicine as people who were stably housed. In a study of 1,626 patients accessing SUD care via telemedicine in Denver (USA), authors found that patients experiencing homelessness were somewhat more likely to miss a telemedicine encounter than their stably housed counterparts (37% vs. 25%) [103]. A national study on telemedicine-based buprenorphine prescribing for USA veterans reported that retention rates were higher for patients who received at least one video-based visit versus those who only had audio-only visits, and that patients who were homeless or experiencing housing instability (as documented by ICD-10 or clinic codes for homelessness and housing instability) were more likely to only have audio-only visits than those with stable housing [93].

##### 1b) Telemedicine-based SUD counseling

Though some telemedicine-based MOUD prescribing interventions provided concurrent SUD counseling, there were seven additional articles that discussed telemedicine-based SUD counseling interventions without a direct medication prescribing component [82, 85, 86, 88, 97, 99, 114]. For example, in a qualitative study with 36 PEH and homeless services staff across England and Scotland, participants reported that telemedicine-based services offered flexibility and increased engagement in services compared to before telemedicine-based services were available [82].

A few studies reported challenges to telemedicine-based SUD counseling for PEH. For example, a New York City (USA) study that compared telemedicine-visit outcomes by housing status found that patients with unstable housing (including homelessness and transitional housing) had fewer telemedicine counseling visits than stably housed patients [99]. In another New York City (USA) study, among patients who received an in-hospital addiction consultation, only 8% of homeless patients were able to be reached by telephone for ongoing counseling and referrals after their hospitalization [97]. However, fears about technological barriers faced by PEH were not always realized: in a qualitative study in Edinburgh (Scotland), some staff participants noted fears of losing touch with clients because of lost telephones but ultimately found most clients remained contactable via telephone [86].

##### 1c) Mobile unit and street outreach-based MOUD induction and maintenance

Seven articles discussed mobile unit and outreach-based MOUD interventions during the pandemic [54, 58, 62, 67, 73, 107, 116]. Articles suggested that mobile unit-based buprenorphine prescribing seemed to increase access to treatment for PEH. For example, one article described a mobile medical unit staffed with nurses, physicians, pharmacists, and outreach support workers that provided buprenorphine induction, maintenance, and dispensing for underserved populations in Chicago (USA); the authors reported that over 200 individuals (of 587 total patients served) visited the unit for buprenorphine prescriptions or maintenance throughout the first year of operation [62]. In an article describing a different mobile van-based buprenorphine induction program for PEH in Chicago (USA), in which the van’s staff connected patients to buprenorphine providers via telemedicine, authors reported anecdotally that “dozens” of PEH accessed buprenorphine in this manner in the first two months of the program [58]. Neither of these articles reported adverse events associated with mobile unit-based buprenorphine programs.

Articles reporting on other direct outreach interventions to PEH on the streets or in encampments similarly noted successes in connecting PEH to SUD treatment. One article describing outreach-based SUD treatment for residents of homeless encampments in St Louis (USA)—in which a team consisting of a social worker, nurses, a physician, and a peer support worker visited encampments multiple times weekly—reported that at least 35 patients were stabilized on MOUD between March 2020 and January 2021 [67]. Another article describing a new outreach-based buprenorphine prescribing model in Ithaca (USA) reported initiating seven PEH on buprenorphine between March and June 2020 [73]. These articles did not provide data on other patient-level outcomes.

##### 1d) Buprenorphine and methadone provision in pandemic homeless services settings

Twenty-one articles discussed interventions involving onsite buprenorphine and methadone provision, including through prescribing, delivery or onsite dispensing, at I&Q facilities [51–53, 55, 56, 64, 94, 95, 105], protective hotels for PEH [49, 84, 92, 96, 111–113], shelters [61, 71, 98, 102], and a tiny shelter encampment [59]. One additional article described SUD, including OUD, treatment in protective hotels but did not specify treatment type [57]. Articles described successes in MOUD initiation, connections to care, and adherence. For example, in a study in Berlin (Germany), 17% (n = 23) of 139 patients at an I&Q facility for PEH received MOUD via onsite provision, over half of whom had been newly started on MOUD at the I&Q facility [95]. A study of protective hotel residents (n = 259) in Chicago (USA) reported that, of the 89 patients with SUD, 24 with existing MOUD prescriptions successfully received daily doses of buprenorphine or methadone onsite and 9 patients were newly initiated on buprenorphine (also supplied daily onsite) [96]. A case series describing a buprenorphine prescription and delivery program in shelter-in-place hotels for PEH in San Francisco (USA) described three patients who were successfully connected to care [111], and a separate mixed methods study of a buprenorphine delivery program in San Francisco (USA) protective hotels reported that buprenorphine prescriptions increased from 4 to 9% among 346 residents [113]. In an article describing an addiction consult service in an I&Q unit in Sydney (Australia) that served 58 patients, authors reported that 18 patients were continued on MOUD and 11 patients initiated new MOUD treatment; nine of these patients were PEH and others were unstably housed [64]. One case series also suggested that a methadone delivery program at protective hotels for PEH in Hamilton (Canada) helped prevent missed doses and resulted in less illicit drug use for two patients who had previously been prescribed methadone [112].

##### 1e) Methadone take-home dosing and low-barrier provision

Thirteen articles discussed expansion of methadone take-home doses (THDs) [53, 63, 75, 77–79, 82, 85, 87–90, 116], which was facilitated by policies that relaxed THD requirements and allowed providers more freedom to use their discretion in offering THDs during the COVID-19 pandemic [53, 63, 75, 77, 79, 82, 87, 89, 90]. These articles generally reported that expanded methadone THDs were associated with increased feelings of control and autonomy among patients. Two qualitative studies—one including 20 PEH receiving methadone THDs while staying at protective hotels in California (USA) and the other including 196 individuals across Canada (25% of whom were PEH or unstably housed)—found that THDs improved convenience and the sense of control felt by patients [87, 90]. Another qualitative study, which included 22 unstably housed individuals who used drugs in Bristol (England), similarly found that THDs increased patient autonomy and reduced potential for embarrassment associated with daily retrievals [79]. Another qualitative study, which included interviews with people who used drugs and drug treatment providers across England and Scotland, also noted that THDs decreased potentially negative effects associated with daily retrievals, like embarrassment or theft of the medications, and increased patients’ feelings of autonomy [82]. In addition to the studies on methadone THDs, one study examined a unique low-threshold clinic in Hamburg (Germany), in which patients (n = 84, 54% PEH) could receive methadone without health insurance, physical examinations, or blood tests; authors reported a six-month retention rate of 61% [104].

Studies also reported some challenges associated with methadone THDs, especially for PEH. Participants from the aforementioned qualitative studies in Bristol (England) and across England and Scotland reported that extended methadone THDs could disrupt valuable routines related to the daily retrieval of doses [79, 82]. Qualitative study participants in Boston (USA) (n = 10 patients on methadone, 60% of whom were unstably housed) similarly reported a destabilizing impact of THDs, and some reported that their unhoused status meant that they could not access THDs because they lacked a safe place to store medications [78]. Notably, the participants in Boston (USA) who reported these negative experiences were all PEH or had been recently housed, whereas housed participants largely reported that THDs were “liberating” [78].

##### 1f) Treatment for alcohol use disorder

A few articles discussed medication treatment for AUD [51, 54, 56, 96] at I&Q sites [51, 56], protective hotels [96] and via telemedicine [54]. However, most of these articles mentioned AUD treatment amidst treatment for other SUDs, and did not provide details about the specifics of AUD treatment. An article describing a low-barrier telephone consultation program in Los Angeles (USA) reported 28 naltrexone prescriptions out of 662 total medications prescribed, though due to data limitations it is not clear whether these prescriptions were for AUD or OUD [54].

##### 1g) Benzodiazepine maintenance treatment

One article discussed lowered barriers to benzodiazepine maintenance treatment in Dublin (Ireland) as a result of COVID-19 pandemic-related regulatory and practice change [63]. The authors noted that over 70 patients with benzodiazepine use disorders started benzodiazepine maintenance treatment at I&Q and protective hotels, with initial goals of preventing withdrawal; however, given improvements observed in client health and behavior, the healthcare organization shifted to offering benzodiazepines as maintenance treatment for individuals with benzodiazepine dependence [63].

#### 2) Harm reduction interventions

In this section, we discuss articles that described interventions focused on harm reduction services, including those aimed to prevent overdose and withdrawal from drugs or alcohol. These interventions included: (2a) managed alcohol programs (MAPs) (n = 9 articles); (2b) alcohol withdrawal management (n = 8); (2c) supervised consumption services (SCS) (n = 4); and (2d) safer supply prescribing (n = 5).

##### 2a) Managed alcohol programs (MAPs)

Nine articles discussed seven MAPs created during the pandemic, in which set doses of alcohol were provided to patients in I&Q sites [51, 64, 66, 76, 94, 95, 105] and protective hotels [66, 84, 113]. Articles described MAPs located in Australia [64], Germany [95], England [84], Canada [51, 105], and USA [66, 76, 94, 113]. Stated reasons for creating MAPs included preventing alcohol withdrawal [64, 66, 76, 105], enabling individuals to isolate or quarantine [64, 66, 76, 95, 105], and ultimately preventing COVID-19 infection [76, 105]. Apart from one MAP located at an I&Q site in Australia, which was open to housed individuals as well as PEH [64], all MAPs were targeted specifically to PEH.

Articles reported high MAP utilization rates, suggesting that the MAPs were acceptable to patients. Studies of MAPs in I&Q programs in Halifax (Canada) and Berlin (Germany) reported that approximately half of the I&Q clients received alcohol [95, 105]. An article describing a MAP at protective and I&Q hotels for PEH in San Francisco (USA) reported that 26 patients were enrolled in the program within the first two months of operation [66].

Articles suggested that MAPs were effective in preventing alcohol withdrawal and hospitalization and resulted in few adverse events. For example, articles describing I&Q site MAPs in Sydney (Australia) and Juneau (USA) reported that no patients experienced severe alcohol withdrawal or withdrawal requiring hospitalization [64, 76]. Articles describing MAPs at I&Q sites in Halifax (Canada) and San Francisco (USA) similarly reported rare or no adverse events related to the MAP [66, 105].

##### 2b) Alcohol withdrawal management

Eight articles discussed other (non-MAP) interventions related to alcohol withdrawal prevention or management that were newly created or modified during the pandemic [50—52, 55, 60, 64, 80, 91]. We included these articles in the harm reduction category because the articles often discussed how the goal of these withdrawal management programs was to minimize the harm that would come from people leaving protective and I&Q sites early due to alcohol withdrawal. Alcohol withdawal prevention and management programs included outpatient benzodiazepine tapers [52], reduced restrictions around alcohol consumption at a protective hotel [80] and congregate shelters [60], and pharmacological withdrawal management (e.g., with benzodiazepines) at I&Q sites [51, 64] and emergency shelters [50, 91]. Pharmacological withdrawal management at I&Q sites and emergency shelters appeared to be acceptable to patients and effective at preventing withdrawal. One study (n = 69) of a novel low-threshold withdrawal management and adverse effect mitigation program in an emergency congregate shelter in Lisbon (Portugal)—in which residents could access medications (diazepam, thiamine, pyridoxine, folic acid, and cobalamin) without an exam or abstinence from alcohol and were voluntarily referred to a specialized alcohol medical appointment—reported that 36% of residents with self-reported alcohol-related problems decided to begin the pharmacological intervention and that 23% accepted the appointment; participation in the program was linked to improved housing outcomes [91]. An article describing a program in Sydney (Australia) reported that 8 patients at an I&Q site were prescribed benzodiazepines to manage alcohol withdrawal and there were no instances of severe alcohol withdrawal requiring referral to a higher level of care; this site also offered a MAP, which is discussed above [64].

##### 2c) Supervised consumption services (SCS)

Four articles discussed SCS or similar interventions for PEH that began during the pandemic and allowed people to use drugs under the supervision of individuals trained in overdose response. These SCS were located at congregate shelters and an I&Q site in Canada [51, 83, 98, 115]. Articles described several reasons for implementing SCS: (1) an understanding that harm reduction programming, including SCS, was needed to ensure adherence to social distancing recommendations and to prevent COVID-19 infections [51, 98], and (2) to mitigate the risks associated with the toxic drug supply [83, 98, 115].

Articles reported that SCS prevented overdose. In a study of a safer use space (SUS) for use of prescribed safer supply located in a shelter in Hamilton (Canada), researchers reported a significant reduction in non-fatal overdoses, from a rate of 0.93 per 100 nights of shelter bed occupancy prior to SUS operation to a rate of 0.17 per 100 nights after the SUS was introduced [98]. A mixed methods study of an overdose prevention site (OPS) located in a congregate shelter in Edmonton (Canada) found that while there were 66 overdoses at the site, there were no deaths [115]. An article describing an SCS at an I&Q site for PEH in Toronto (Canada) reported no fatal overdoses among witnessed drug use at the site – the four fatal overdoses at the I&Q site were unwitnessed (i.e., not occurring at the SCS) [51].

Additionally, articles reported that SCS were widely used and responded to a large number of overdoses. For example, articles reported that a SCS at a congregate shelter in Kingston (Canada) responded to over 400 overdoses in the first year it had been open [83] and the OPS in Edmonton (Canada) was accessed 1346 times by 174 individuals in the two months it was open. Users of the Edmonton (Canada) OPS also reported that it was convenient, safe, accessible, and reduced their drug use in other parts of the shelter [115]. None of the articles reported adverse effects.

##### 2d) Safer supply prescribing

Five articles discussed the creation of safer supply interventions, in which pharmaceutical-grade medications were provided as alternatives to the illicit drug supply, that served PEH. Three interventions involved prescription of hydromorphone tablets [51, 52, 98]; one prescribed hydromorphone, morphine, and dextroamphetamine [108]; and one prescribed hydromorphone, prescription stimulants, and benzodiazepines [105]. All safer supply interventions were in Canada; one was shelter-based [98], one was hospital-based [52], and three were located at I&Q hotels for PEH [105, 51, 108]. The articles noted that safer supply interventions aimed to prevent COVID-19 infections through enabling adherence to social distancing [52, 98, 105, 108] and to mitigate the risks of overdose amidst a toxic unregulated drug supply [52, 105, 108].

The articles suggested that safer supply interventions were acceptable and utilized by patients. For example, a case series of 77 residents of an I&Q hotel for PEH in Nova Scotia (Canada) that offered safer supply prescribing reported that 27 residents were prescribed hydromorphone tablets, 31 were prescribed stimulants, and six were prescribed benzodiazepines [105]. A case report of a patient prescribed hydromorphone and dextroamphetamine in an I&Q hotel in British Columbia (Canada) reported that the safer supply prescribing allowed the individual to remain in isolation, reduce illicit drug use, and connect to healthcare [108]. Safer supply interventions also appeared to be safe, with few reported adverse effects. One article reported that there were no overdoses and that medication diversion or sharing only occurred three times over 1059 person-days of isolation [105]. The other articles did not report adverse events related to the safer supply prescribing.

## Discussion

This scoping review identified 68 peer-reviewed articles about substance use-related interventions for PEH that either arose newly or changed substantially in response to the COVID-19 pandemic. This review gives a timely overview of innovations that address the needs of PEH that are practicable even beyond the pandemic context. Examining service innovations is particularly important amidst rising rates of homelessness [117] and an ongoing overdose crisis post-pandemic that has become increasingly concentrated among highly marginalized populations, including PEH [118–121].

Several articles in this review shed light on telemedicine-based SUD services implemented during the COVID-19 pandemic, which appeared to have benefits including increased access to treatment [75, 107] and reduced barriers to care [81]. While some PEH faced challenges related to telemedicine, including lack of access to telephones, inconsistent telephone numbers, and other technological barriers [52, 75, 69, 100], articles in this review also described innovative measures to address these challenges. These mitigation measures included facilitated telemedicine (e.g., outreach workers using their telephones or tablets to connect patients in the field with offsite providers) [65, 70, 75, 107, 109], audio-only telemedicine [106, 100, 74], and pre-paid telephone provision [101]. Other articles described mobile-unit and other outreach interventions that used integrated telemedicine; these also appeared to be successful in connecting PEH to buprenorphine [58, 73]. Such measures to enhance accessibility to telemedicine may also be applicable to other populations apart from PEH, for whom similar challenges have been reported [122].

Articles in this scoping review also offered insights on changes in methadone provision, which, at least in the USA, had been largely provided via entrenched models of daily supervised dosing at specialty clinics [123–125]. First, articles suggested that onsite methadone provision (including via methadone delivery) at pandemic homeless services settings, including emergency shelters, protective hotels, and I&Q sites, helped connect PEH to treatment and promoted adherence [64, 112]. Second, articles examining changes in methadone THD provision suggested that THDs were acceptable to patients [79, 82, 87], although the potential for treatment destabilization was identified as a possible limitation [78, 79, 82]. Other research evaluating outcomes associated with methadone THD more broadly has shown that methadone-involved overdoses did not increase amidst COVID-19 pandemic-related methadone THD policy changes, despite an overall increase in all overdose deaths during that time [126, 127].

Additionally, several articles in this scoping review demonstrated the safety and promise of various harm reduction interventions including SCS [51, 83, 98, 115], MAPs [66, 76, 105], and safer supply prescribing [98, 105, 108] for PEH. MAPs and safer supply interventions applied in pandemic settings, such as I&Q settings or protective hotels, allowed PEH to successfully complete their recommended duration of stays (e.g., in isolation or quarantine) or remain in protective settings for longer periods [66, 76, 105, 108]. The apparent successes of the onsite delivery of these harm reduction interventions suggest that these harm reduction interventions may have potential applications in other emergency response contexts, or more generally in clinical or service settings where PEH may otherwise self-discharge due to inadequate management of substance dependence and withdrawal.

The literature on novel interventions such as SCS and safer supply used during the COVID-19 pandemic has continued to evolve since the time of our literature search for this scoping review, with successes being reported globally [128–130]. For example, a recent study in Toronto (Canada) demonstrated the feasibility and acceptability of implementing safer supply at a COVID-19 I&Q site for PEH [128]. Additionally, an evaluation of a safer supply prescribing program (referred to as Risk Mitigation Guidance or RMG dispensations) implemented across British Columbia (Canada) during the COVID-19 pandemic showed that receiving RMG opioid dispensations for one day or more was associated with a reduced likelihood of all-cause and overdose-related mortality in the subsequent week [130]. A mixed methods study investigating the implementation of a drug consumption room (a form of SCS) in Athens (Greece) during the COVID-19 pandemic reported approximately 11,000 visits in the first 6 months of operation [129]. These recent studies further reflect the breadth of harm reduction innovations that emerged during the COVID-19 pandemic, and the value they may have beyond the pandemic context.

Despite the promise of the innovations described in this review, there is a need for further research to build on these findings. First, much of the research was observational in nature, which limits the ability to draw causal inference and fully assess intervention impacts. Future studies should rigorously evaluate intervention effectiveness using robust study designs including randomized controlled trials or quasi-experimental designs with control groups where possible. Future research should also examine any differential impacts of interventions—such as those related to onsite methadone access and telemedicine-based MOUD prescribing—for PEH compared to other populations. Many studies have included PEH among other intervention recipients but have not looked specifically at outcomes for PEH, which limits our understanding of the unique barriers and opportunities these interventions might pose for PEH. Finally, comparative or cross-country research could further investigate substance use and harm reduction interventions for PEH across different policy contexts that might impact the implementation and efficacy of different interventions.

Adaptions and innovations introduced during the COVID-19 pandemic have the potential to guide lasting change and this scoping review has important policy implications. For example, lower-barrier, more flexible approaches to MOUD provision showed significant promise with few reported adverse effects, and their continuation may help reduce barriers to treatment [131]. It is encouraging that the USA has made permanent the easing of regulations for methadone THD introduced during the pandemic, meaning patients can now receive up to 28 days of medication after 1 month in treatment [132]. However, practicalities inherent in homelessness, such as challenges in safely storing medications like methadone, may limit the impact of this regulation specifically for PEH [78]. Also, in the USA, while buprenorphine can be prescribed by medical providers and dispensed in retail pharmacies, regulations still prohibit methadone dispensing outside of highly regulated opioid treatment programs [132]. Articles included in this scoping review suggested that onsite MOUD provision, including buprenorphine prescribing and methadone dispensing or deliveries within settings serving PEH, may have improved treatment uptake and adherence during the pandemic by confronting usual logistical challenges and access barriers [64, 95, 96, 111–113]. Future research should examine the post-pandemic feasibility and effectiveness of policies to facilitate MOUD provision in more accessible settings to minimize treatment barriers faced by PEH. Articles included in this scoping review also suggested that integrating robust harm reduction practices such as SCS and safe supply prescribing into homeless services settings helped to mitigate overdose risks [51, 83, 98, 105, 108, 115]. As many parts of the world continue to have legislative and regulatory barriers to implementation of SCS and safer supply prescribing, the evidence presented in this review could strengthen the evidence base for policymakers considering implementing or expanding access to these services. Finally, as a fundamental determinant of health, the biggest policy imperative remains addressing homelessness itself. Without investment and political commitment to expand permanent, affordable housing options, interventions aiming to improve substance use and other health outcomes among PEH may have a limited impact.

### Limitations

There are several limitations of this review. As the intent of this review was to provide an overview of the breadth and variety of novel substance use-related interventions for PEH that arose during the COVID-19 pandemic, the inclusion criteria were broad. Therefore, articles with a range of methodologies and populations were included, including many that were descriptive program summaries without any analytical data. Due to this heterogeneity, a meta-analysis was not feasible. More generally, conclusions that can be drawn regarding effectiveness of the described interventions are limited by the methodologic rigor of the included studies. Not surprisingly given the pandemic context, our literature search revealed no randomized trials and very few studies that included any sort of comparison group, limiting the ability to draw causal inference.

Moreover, we limited our search to articles published in English, potentially limiting the geographic scope of the included articles. Our final database search was conducted in September 2023, so articles published more recently are not included [133, 134]. Our review was limited to published articles or reports. Innovative and important interventions led by people and organizations lacking the capacity to conduct research or write reports during the pandemic would be missing from the review. Though we worked with a research librarian on best practices for grey literature searching, our search of the grey literature returned no additional relevant results. We did not perform an updated search of the grey literature after the initial search conducted in November 2022, as our initial grey literature search did not yield any relevant articles beyond the academic articles already found in our database searches. We do not anticipate that we would have found anything critical not found in the academic database search. Despite these limitations, this scoping review provides a comprehensive snapshot of new interventions related to substance use for PEH that were implemented during the COVID-19 pandemic.

## Conclusions

This scoping review uncovered diverse ways in which providers and policy makers crea`ted and adapted programs to deliver substance use treatment and harm reduction services for PEH during the COVID-19 pandemic. Many programs leveraged changing regulations and new urgency spawned by a desire to prevent COVID-19 infections to increase access to substance use and harm reduction services. Evidence from the included articles suggests that novel or underutilized harm reduction interventions, such as MAPs, SCS, and safer supply, were feasible to implement for PEH, with few, if any, adverse effects described. PEH faced some unique barriers to telemedicine interventions, but articles in this review described creative solutions to facilitate access. Other interventions to reduce barriers to substance use treatment for PEH such as onsite methadone provision and mobile outreach-based MOUD prescribing showed promise and should be further studied. Particularly as PEH continue to face high rates of overdose death [118–121], it is important to implement interventions that consider the unique challenges faced by PEH. Future research should investigate how to best implement and sustain effective innovations to reduce drug-related harms and barriers to substance use treatment and harm reduction services faced by PEH post-pandemic.

## Supplementary Information

Supplementary material 1: Appendix A. Search strategy; search strategy for database searches.

Supplementary material 2: Appendix B. Data extraction template; template used for data extraction.

Supplementary material 3: Appendix C. List of excluded full texts and reasons for exclusion (n = 80); list of all texts that were screened and excluded at the full text level with reasons for exclusion.

## Data Availability

No datasets were generated or analysed during the current study.

## Abbreviations

AUD: Alcohol use disorder
COVID-19: Coronavirus Disease 2019
I&Q: Isolation and quarantine
MAP: Managed alcohol program
MOUD: Medications for opioid use disorder
PEH: People experiencing homelessness
SCS: Supervised consumption services
SUD: Substance use disorder
THD: Take-home dose
UK: United Kingdom
USA: United States of America
