# Supplementary material for: Innovations at the intersection of homelessness and substance use during the COVID-19 pandemic: a scoping review
Source: Harm Reduct J. 2025 Jul 29;22:132. doi: 10.1186/s12954-025-01235-7 (PMC12308940; doi:10.1186/s12954-025-01235-7)
Supplement: Supplementary file 1 — Supplementary material 1: Appendix A. Search strategy; search strategy for database searches. [file 12954_2025_1235_MOESM1_ESM.docx]

**Appendix A. Search strategy**

| **Database** | **Search terms**  **(formatted based on database requirements)** | **Filters applied, if any** |
| --- | --- | --- |
| Embase | ("substance-related disorders" or ("substance-related" and "disorders") or "substance use" or ("substance" and "use") or "substance abuse" or ("substance" and "abuse") or "drug abuse" or ("drug" and "abuse") or "addict" or "addicted" or "addiction" or "addicts" or "substance misuse" or ("substance" and "misuse") or "alcohol use" or ("alcohol" and "use") or "alcohol drinking" or ("alcohol" and "drinking") or "alcoholic" or "alcoholism") and ("homeless" or "homelessness" or "homeless persons" or ("homeless" and "persons") or "homeless youth" or ("homeless" and "youth") or "housing") and ("COVID-19" or "SARS-CoV-2" or "sars-cov-2" or "NCOV" or "2019 NCOV" or "coronavirus") | Publication date 2020 and later; English language |
| PubMed | (substance use OR substance abuse OR drug abuse OR "drug use" OR addiction OR alcohol drinking OR alcoholism OR ethanol) AND (homelessness OR housing) AND (COVID-19) | Publication date 2020 and later; English language |
| PsycInfo | ("substance-related disorders" or ("substance-related" and "disorders") or "substance use" or ("substance" and "use") or "substance abuse" or ("substance" and "abuse") or "drug abuse" or ("drug" and "abuse") or "addict" or "addicted" or "addiction" or "addicts" or "substance misuse" or ("substance" and "misuse") or "alcohol use" or ("alcohol" and "use") or "alcohol drinking" or ("alcohol" and "drinking") or "alcoholic" or "alcoholism") and ("homeless" or "homelessness" or "homeless persons" or ("homeless" and "persons") or "homeless youth" or ("homeless" and "youth") or "housing") and ("COVID-19" or "SARS-CoV-2" or "sars-cov-2" or "NCOV" or "2019 NCOV" or "coronavirus") | Publication date 2020 and later; English language |
| Web of Science | (substance-related disorders or (substance-related and disorders) or substance use or (substance and use) or substance abuse or (substance and abuse) or drug abuse or (drug and abuse) or addict or addicted or addicts addiction or substance misuse or (substance and misuse) or alcohol use or (alcohol and use) or alcohol drinking or (alcohol and drinking) or alcoholic or alcoholism) and (homeless or homelessness or homeless persons or (homeless and persons) or homeless youth or (homeless and youth) or housing) and (COVID-19 or SARS-CoV-2 or sars-cov-2 or NCOV or 2019 NCOV or coronavirus) | Publication date 2020 and later; English language |
| CINAHL | ( (substance-related disorders or (substance-related and disorders) or substance use or (substance and use) or substance abuse or (substance and abuse) or drug abuse or (drug and abuse) or addict or addicted or addicts addiction or substance misuse or (substance and misuse) or alcohol use or (alcohol and use) or alcohol drinking or (alcohol and drinking) or alcoholic or alcoholism) ) AND ( (homeless or homelessness or homeless persons or (homeless and persons) or homeless youth or (homeless and youth) or housing) ) AND ( (COVID-19 or SARS-CoV-2 or sars-cov-2 or NCOV or 2019 NCOV or coronavirus) ) | Expanders - Apply related words; Apply equivalent subjects  Limiters - Publication date 2020 and later; English language |
| OAlster | kw:(substance use OR substance abuse OR drug use OR drug abuse OR addiction OR alcoholism) AND kw:(homelessness OR homeless OR housing) AND kw:(covid-19) |  |
| NYAM | (substance use OR substance abuse OR drug abuse OR drug use OR addiction OR alcohol drinking OR alcoholism OR ethanol) AND (homelessness OR housing) AND COVID-19 |  |
| OpenGrey | (substance use OR substance abuse OR drug use OR drug abuse OR addiction OR substance related disorder OR substance misuse OR alcohol use OR alcohol abuse OR alcohol misuse OR alcoholism) AND (homelessness OR homeless OR homeless person OR housing) AND COVID-19 |  |
| Google | (substance use \| substance abuse \| drug use \| drug abuse \| addiction \| substance use disorder \| substance misuse \| alcohol use \| alcohol abuse \| alcohol misuse \| alcoholism) AND (homelessness \| homeless \| housing) AND (COVID \| coronavirus) | Feb 1, 2020-date of search, English language |
