# Supplementary material for: Innovations at the intersection of homelessness and substance use during the COVID-19 pandemic: a scoping review
Source: Harm Reduct J. 2025 Jul 29;22:132. doi: 10.1186/s12954-025-01235-7 (PMC12308940; doi:10.1186/s12954-025-01235-7)
Supplement: Supplementary file 2 — Supplementary material 2: Appendix B. Data extraction template; template used for data extraction. [file 12954_2025_1235_MOESM2_ESM.docx]

**Appendix B. Data extraction template**

Data extraction was conducted in the review software Covidence. Data from each included study was extracted by one study team member and then checked for accuracy by an additional team member. This template includes all of the response categories and response options, where applicable.

**General information**

1. Study ID
2. Title
   1. Title of paper / abstract / report that data are extracted from
3. Country in which the study was conducted
   1. United States
   2. UK
   3. Canada
   4. Australia
   5. Other:
4. City in which study was conducted
5. Type of location
   1. Urban
   2. Rural
   3. Suburban
   4. Other:
6. Setting
   1. Hospital
   2. Hotel
   3. Non-clinic outpatient treatment facility
   4. Clinic
   5. Community
   6. Recovery home
   7. Shelter
   8. Other:
7. Purpose of study
8. Rationale for why it was done

**Characteristics of included studies**

1. Methods
   1. Study design
      1. Randomized control trial
      2. Non-randomized experimental study
      3. Cohort study
      4. Cross sectional study
      5. Case control study
      6. Systematic review
      7. Qualitative research
      8. Prevalence study
      9. Case series
      10. Case report
      11. Diagnostic test accuracy study
      12. Clinical prediction rule
      13. Economic evaluation
      14. Text and opinion
      15. Other:
   2. Start date of study period/intervention
   3. End date of study period/intervention
2. Participants
   1. Population involved
   2. Total number of participants
   3. Number who received intervention
   4. Number who did not receive intervention
   5. Portion of population unhoused
   6. Portion of population with SUD
   7. Baseline population characteristics

|  | # of participants | % of participants |
| --- | --- | --- |
| Male |  |  |
| Female |  |  |
| Housed |  |  |
| Unhoused |  |  |
| Age range |  |  |
| White or Caucasian |  |  |
| Black |  |  |
| Hispanic or Latinx |  |  |
| Asian or Pacific Islander |  |  |
| Native American |  |  |
| Multiracial or multiethnic |  |  |

1. Intervention
   1. What was the intervention?
   2. Duration of intervention, for individual participants
   3. Substance use addressed by intervention
      1. Alcohol
      2. Nicotine
      3. Opioids
      4. Marijuana
      5. Other:
   4. Type of intervention
      1. Provision of temporary shelter
      2. Medications for SUD
      3. Provision of non-SUD medications
      4. Managed alcohol supply
      5. Safe injection site
      6. Safe consumption supplies (syringes, needles, etc.)
      7. Appointments with SUD healthcare providers
      8. SUD counseling
      9. Overdose prevention (naloxone, etc.)
      10. Other:
   5. Change made to intervention due to COVID
      1. Increased dose
      2. Decreased time/frequency of appointments
      3. Moved to virtual/telehealth
      4. Expanded access/decreased eligibility requirements
      5. Was created
      6. Other:
2. Results
   1. Method of collecting results
   2. Primary outcome
   3. Results for primary outcome
   4. Secondary outcome(s)
   5. Results for secondary outcome(s)
   6. SUD related results/successes
   7. Concerns or potential challenges
   8. Adverse outcomes
   9. Insights for subsequent SUD interventions in homeless populations
   10. Insights for future pandemics

**Final steps**

1. Reference screen: any relevant references not already included?
   1. No; all references irrelevant/already included
   2. Yes; uploaded to Covidence
   3. Other:
2. Team communication – notes for other team members (include name)
