# Supplementary material for: Innovations at the intersection of homelessness and substance use during the COVID-19 pandemic: a scoping review
Source: Harm Reduct J. 2025 Jul 29;22:132. doi: 10.1186/s12954-025-01235-7 (PMC12308940; doi:10.1186/s12954-025-01235-7)
Supplement: Supplementary file 3 — Supplementary material 3: Appendix C. List of excluded full texts and reasons for exclusion (n = 80); list of all texts that were screened and excluded at the full text level with reasons for exclusion. [file 12954_2025_1235_MOESM3_ESM.docx]

**Appendix C: List of excluded full texts and reasons for exclusion (n=80)**

| **Article** | **Reason for exclusion** |
| --- | --- |
| Abbs, E. S., Raganold, E., Lakatos, K. O., Sawires, S., & Zevin, B. (2021). Expanding buprenorphine starts for people experiencing homelessness during COVID. *Journal of Addiction Medicine*, *15*(5), E22. <https://doi.org/10.1097/ADM.0000000000000902> | Abstract only (no full text published) |
| Adams, E. A., Parker, J., Jablonski, T., Kennedy, J., Tasker, F., Hunter, D., Denham, K., Smiles, C., Muir, C., O’Donnell, A., Widnall, E., Dotsikas, K., Kaner, E., & Ramsay, S. E. (2022). A qualitative study exploring access to mental health and substance use support among individuals experiencing homelessness during COVID-19. *International Journal of Environmental Research and Public Health*, *19*(6), 3459. <https://doi.org/10.3390/ijerph19063459> | No intervention / insufficient details about intervention |
| Ahrens, K., Blackburn, N., Aalsma, M., Haggerty, K., Kelleher, K., Knight, D. K., Joseph, E., Mulford, C., Ryle, T., & Tolou-Shams, M. (2023). Prevention of opioid use and disorder among youth involved in the legal system: innovation and implementation of four studies funded by the NIDA HEAL Initiative. *Prevention Science*, *24*(Suppl 1), 99-110. https://doi.org/10.1007/s11121-023-01566-6 | Intervention not in response to COVID-19 pandemic |
| Alavi, M., Moghanibashi-Mansourieh, A., Radfar, S. R., Alizadeh, S., Bahramabadian, F., Esmizade, S., Dore, G. J., Sedeh, F. B., & Deilamizade, A. (2021). Coordination, cooperation, and creativity within harm reduction networks in Iran: COVID-19 prevention and control among people who use drugs. *International Journal of Drug Policy*, *93*, e102908. https://doi.org/10.1016/j.drugpo.2020.102908 | Intervention not about substance use |
| Alexander, K., Nordeck, C. D., Rosecrans, A., Harris, R., Collins, A., & Gryczynski, J. (2023). The effect of a non‐congregate, integrated care shelter on health: A qualitative study. *Public Health Nursing*, *40*(4), 487-496. <https://doi.org/10.1111/phn.13197> | Intervention not about substance use |
| Amram, O., Amiri, S., Thorn, E. L., Lutz, R., & Joudrey, P. J. (2022). Changes in methadone take-home dosing before and after COVID-19. *Journal of Substance Abuse Treatment*, *133*, e108552. <https://doi.org/10.1016%2Fj.jsat.2021.108552> | Doesn't meaningfully address the needs of people experiencing homelessness |
| Bagley, S. M., Hadland, S. E., & Yule, A. M. (2021). A commentary on the impact of COVID-19 on engagement of youth with substance use and co-occurring psychiatric disorders. *Journal of Substance Abuse Treatment*, *121*, e108175. <https://doi.org/10.1016/j.jsat.2020.108175> | Doesn't meaningfully address the needs of people experiencing homelessness |
| Baker, O., Wellington, C., Price, C. R., Tracey, D., Powell, L., Loffredo, S., Moscariello, S., & Meyer, J. P. (2023). Experience delivering an integrated service model to people with criminal justice system involvement and housing insecurity. *BMC Public Health*, *23*(1), 222. <https://doi.org/10.1186/s12889-023-15108-w> | Intervention not in response to COVID-19 pandemic |
| Barbaglia, G., Pasarin, M., Aranda, E., Clotas, C., Garrido, A., Gotsens, M., Parés-Badell, O., & Bartroli, M. (2021). When policy and opportunity meet: Barcelona’s first gender responsive shelter for homeless drug users. *European Journal of Public Health*, *31*, iii563-iii564. <https://doi.org/10.1093/eurpub/ckab165.594> | Abstract only (no full text published) |
| Cales, R. H., Cales, S. C., Shreffler, J., & Huecker, M. R. (2022). The COVID-19 pandemic and opioid use disorder: Expanding treatment with buprenorphine, and combining safety precautions with telehealth. *Journal of Substance Abuse Treatment*, *133*, 108543. <https://doi.org/10.1016/j.jsat.2021.108543> | Doesn't meaningfully address the needs of people experiencing homelessness |
| Campbell, A., Millen, S., Guo, L., Jordan, U., Taylor-Beswick, A., Rintoul, C., & Diamond, A. (2023). Reducing opioid related deaths for individuals who are at high risk of death from overdose: a co-production study with people housed within prison and hostel accommodation during Covid-19. *Frontiers in Public Health*, *11*, e1080629. <https://doi.org/10.3389/fpubh.2023.1080629> | No intervention / insufficient details about intervention |
| Carver, H., Ciolompea, T., Conway, A., Kilian, C., McDonald, R., Meksi, A., & Wojnar, M. (2023). Substance use disorders and COVID-19: reflections on international research and practice changes during the “poly-crisis”. *Frontiers in Public Health*, *11*, e1201967. <https://doi.org/10.3389/fpubh.2023.1201967> | Doesn't meaningfully address the needs of people experiencing homelessness |
| Clifasefi, S. L., Frohe, T., Taylor, E. M., Shinagawa, E., Collins, S. E. (2023) Housing first residents' perspectives on remote service provision of the life enhancing alcohol-management program during the COVID-19 pandemic. *Alcoholism: Clinical and Experimental Research, 47*(Suppl 1), 455-456. <https://doi.org/10.1111/acer.15071> | Abstract only (no full text published) |
| Collins, A. B., Edwards, S., McNeil, R., Goldman, J., Hallowell, B. D., Scagos, R. P., & Marshall, B. D. L. (2022). A rapid ethnographic study of risk negotiation during the COVID-19 pandemic among unstably housed people who use drugs in Rhode Island. *International Journal of Drug Policy*, *103*, e103626. <https://doi.org/10.1016/j.drugpo.2022.103626> | No intervention / insufficient details about intervention |
| Columb, D., Hussain, R., & O’Gara, C. (2020). Addiction psychiatry and COVID-19: impact on patients and service provision. *Irish Journal of Psychological Medicine*, *37*(3), 164-168. <https://doi.org/10.1017%2Fipm.2020.47> | Doesn't meaningfully address the needs of people experiencing homelessness |
| Comeau, E., Bonn, M., Wildeman, S., & Herder, M. (2023). ‘More of the same, but worse than before’: A qualitative study of the challenges encountered by people who use drugs in Nova Scotia, Canada during COVID-19. *PLoS One*, *18*(4), e0283979. <https://doi.org/10.1371%2Fjournal.pone.0283979> | No intervention / insufficient details about intervention |
| Conway, B., Truong, D., & Wuerth, K. (2020). COVID-19 in homeless populations: Unique challenges and opportunities. *Future Virology*, *15*(6), 331-334. <https://doi.org/10.2217/fvl-2020-0156> | Intervention not about substance use |
| Conway, A., Treloar, C., Crawford, S., Degenhardt, L., Dore, G. J., Farrell, M., Hayllar, J., Grebely, J., & Marshall, A. D. (2023). “You'll come in and dose even in a global pandemic”: A qualitative study of adaptive opioid agonist treatment provision during the COVID-19 pandemic. *International Journal of Drug Policy*, *114*, e103998. <https://doi.org/10.1016%2Fj.drugpo.2023.103998> | Doesn't meaningfully address the needs of people experiencing homelessness |
| Crowley, D., & Cullen, W. (2021). Caring for opioid drug users during the COVID-19 pandemic–a commentary on the Irish experience. *Heroin Addiction and Related Clinical Problems*, *23*(3), 77-81. <https://www.drugsandalcohol.ie/32214/> | Doesn't meaningfully address the needs of people experiencing homelessness |
| Dawes, J., May, T., Fancourt, D., & Burton, A. (2022). The impact of the Covid-19 pandemic and associated societal restrictions on people experiencing homelessness (PEH): a qualitative interview study with PEH and service providers in the UK. *International Journal of Environmental Research and Public Health*, *19*(23), 15526. <https://doi.org/10.3390/ijerph192315526> | No intervention / insufficient details about intervention |
| Dawes, J., May, T., Fancourt, D., & Burton, A. (2022). The impact of the COVID-19 pandemic on people experiencing homelessness: a qualitative interview study in the UK. *The Lancet*, *400*(Suppl 1), S35. <https://doi.org/10.1016/S0140-6736(22)02245-0> | No intervention / insufficient details about intervention |
| Deilamizade, A., & Moghanibashi-Mansourieh, A. (2020). Challenges of providing COVID-19 prevention services to homeless people who use drugs in Iran. *International Journal of Drug Policy*, *83*, e102806. <https://doi.org/10.1016%2Fj.drugpo.2020.102806> | No intervention / insufficient details about intervention |
| Divakaran, B., Bloch, N., Sinha, M., Steiner, A., & Shavit, S. (2023). The Reentry Health Care Hub: Creating a California-based referral system to link chronically ill people leaving prison to primary care. *International Journal of Environmental Research and Public Health*, *20*(10), 5806. <https://doi.org/10.3390/ijerph20105806> | Intervention not in response to COVID-19 pandemic |
| Durand, L., Boland, F., Harnedy, N., Delargy, Í., Scully, M., Bourke, M., Ebbitt, W., Vázquez, M. O., Keenan, E., & Cousins, G. (2023). Impact of changes to the delivery of opioid agonist treatment, introduced during the COVID-19 pandemic, on treatment access and dropout in Ireland: An interrupted time series analysis. *Journal of Substance Use and Addiction Treatment*, *149*, e209029. <https://doi.org/10.1016/j.josat.2023.209029> | Doesn't meaningfully address the needs of people experiencing homelessness |
| Friedman, J., Calderón-Villarreal, A., Adame, R. C., Abramovitz, D., Rafful, C., Rangel, G., Vera, A., Strathdee, S. A., & Bourgois, P. (2022). An ethnographic assessment of COVID-19‒related changes to the risk environment for people who use drugs in Tijuana, Mexico. *American Journal of Public Health*, *112*(S2), 199-205. <https://doi.org/10.2105/ajph.2022.306796> | No intervention / insufficient details about intervention |
| Frizelle, F. (2023). Homeless in Aotearoa New Zealand-paradise lost. *The New Zealand Medical Journal (Online)*, *136*(1570), 8-11. <https://doi.org/10.26635/6965.e1570> | No intervention / insufficient details about intervention |
| Frohe, T., Leemon, G., Taylor, E., Hamdy, N., Cohn, E., Davis, A., Fentress, T., Williams, G., Clifasefi, S., & Collins, S. (2022). Content analysis of Housing First residents’ perspectives on service provision during the COVID-19 pandemic. *Alcoholism: Clinical and Experimental Research, 46*(Suppl 1), 163. <https://doi.org/10.1111/acer.14833> | Intervention not about substance use |
| Frost, M. C., Austin, E. J., Corcorran, M. A., Briggs, E. S., Behrends, C. N., Juarez, A. M., Frank, N. D., Healy, E., Prohaska, S. M., & LaKosky, P. A. (2022). Responding to a surge in overdose deaths: perspectives from US syringe services programs. *Harm Reduction Journal*, *19*(1), 79. <https://doi.org/10.1186/s12954-022-00664-y> | Doesn't meaningfully address the needs of people experiencing homelessness |
| Garvin, L. A., Greenan, M. A., Edelman, E. J., Slightam, C., McInnes, D. K., & Zulman, D. M. (2023). Increasing use of video telehealth among veterans experiencing homelessness with substance use disorder: Design of a peer-led intervention. *Journal of Technology in Behavioral Science*, *8*(3), 234-245. <https://doi.org/10.1007/s41347-022-00290-2> | No intervention / insufficient details about intervention |
| Ghosh, A., Naskar, C., Roub, F. E., & Basu, D. (2021). Review of the adaptations in opioid agonist treatment during the COVID-19 pandemic: focus on buprenorphine-based treatment. *Journal of Opioid Management*, *17*(7), 119-131. <https://doi.org/10.5055/jom.2021.0649> | Review article |
| Hagle, H., Sung, M., Drexler, K., Waters, E., Becker, W., Blaney-Koen, D., Blevins, D., Edelman, E. J., Molfenter, T., & Cates-Wessel, K. (2021). Clinicians' response to COVID-19: Impact on clinical practice and policies in treating opioid use disorders (MOUD). *The American Journal on Addictions, 30*(3), 240–285. <https://doi.org/10.1111/ajad.13173> | Doesn't meaningfully address the needs of people experiencing homelessness |
| Holmes, R. P., Rezk, T., Lang, P. C., Sanchez, L., Morales, J., Navarro, J., Tarkanian, B. COVID-19 response in an urban safety net treatment program. *Journal of Addiction Medicine 15*(5), e12. <https://doi.org/10.1097/ADM.0000000000000902> | Intervention not about substance use |
| Johnson, A., Goldenberg, S., Worley, S., Shen, M., Lan, Y., Isaacs, N., Philippou, C., Karim, E., Beaubrun, J., Blissett, K. A., Balaban, M., Keeley, C., & Long, T. G. (2023). Expanding Harm Reduction & Oud Treatment Options by Combining Mobile Health & Telemedicine. *Journal of General Internal Medicine*, *38*(Suppl 2), S704-S705. <https://dx.doi.org/10.1007/s11606-023-08226-z> | Abstract only (no full text published) |
| Julie, M. D. (2022). Recidivism and Offenders with Serious Mental Illnesses. *University of Arizona Global Campus ProQuest Dissertations & Theses.* <https://www.proquest.com/openview/c061c2b0b951578f4fdaea9b876e994e/1?pq-origsite=gscholar&cbl=18750&diss=y> | Intervention not in response to COVID-19 pandemic |
| Kalofonos, I., & McCoy, M. (2023). Purity, danger, and patriotism: The struggle for a veteran home during the COVID-19 pandemic. *Pathogens*, *12*(3), 482. <https://doi.org/10.3390/pathogens12030482> | No intervention / insufficient details about intervention |
| Kanter, K., Gallagher, R., Eweje, F., Lee, A., Gordon, D., Landy, S., Gasior, J., Soto-Calderon, H., Cronholm, P. F., Cocchiaro, B., Weimer, J., Roth, A., Lankenau, S., & Brenner, J. (2021). Willingness to use a wearable device capable of detecting and reversing overdose among people who use opioids in Philadelphia. *Harm Reduction Journal*, *18*(75). <https://doi.org/10.1186/s12954-021-00522-3> | No intervention / insufficient details about intervention |
| Kedia, S. K., Schmidt, M., Dillon, P. J., Arshad, H., & Yu, X. (2021). Substance use treatment in Appalachian Tennessee amid COVID-19: Challenges and preparing for the future. *Journal of Substance Abuse Treatment*, *124*, e108270. <https://doi.org/10.1016/j.jsat.2020.108270> | No intervention / insufficient details about intervention |
| Kelly, E. L., Reed, M. K., Schoenauer, K. M., Smith, K., Scalia-Jackson, K., Kay Hill, S., Li, E., & Weinstein, L. (2022). A qualitative exploration of the functional, social, and emotional impacts of the COVID-19 pandemic on people who use drugs. *International Journal of Environmental Research and Public Health*, *19*(15), 9751. <https://doi.org/10.3390/ijerph19159751> | No intervention / insufficient details about intervention |
| Kennedy, A. J., Rossetti, G., Chavez, M., Angelo, N., & Hurley, B. (2022). Providing low-barrier medications for addiction treatment via a telemedicine call line to people experiencing homelessness is Los Angeles, County. *Journal of General Internal Medicine, 37*(Suppl 2)*,* 163-163. <https://doi.org/10.1007/s11606-022-07653-8> | Updated version of the same article available for review |
| Knopf, A. (2020). Homelessness, mental illness, COVID‐19 and SUD handled by Hawaii BHA. *Alcoholism & Drug Abuse Weekly*, *32*(43), 1-4. <https://doi.org/10.1002/adaw.32886> | No intervention / insufficient details about intervention |
| Krawczyk, N., Allen, S. T., Schneider, K. E., Solomon, K., Shah, H., Morris, M., Harris, S. J., Sherman, S. G., & Saloner, B. (2022). Intersecting substance use treatment and harm reduction services: exploring the characteristics and service needs of a community-based sample of people who use drugs. *Harm Reduction Journal*, *19*(1), 95. <https://doi.org/10.1186/s12954-022-00676-8> | Doesn't meaningfully address the needs of people experiencing homelessness |
| Labriola, M. M., Sobol, D., Sims, H., & Holliday, S. B. (2023). Implementation and outcome evaluation of LA DOOR: A Proposition 47-funded program in Los Angeles: Cohort 2 final evaluation report. *Rand Health Quarterly*, *10*(4). <https://www.rand.org/pubs/research_reports/RRA1500-2.html> | Intervention not in response to COVID-19 pandemic |
| Letcher, A., Cuadrado, H. M., Harbove, S., & Taveras, Y. J. (2021). Developing capacity for low barrier access through community partnerships during COVID-19. *Journal of Addiction Medicine*, *15*(5), E13-E14. <https://doi.org/10.1097/ADM.0000000000000902> | Abstract only (no full text published) |
| Levander, X. A., Couch, J., Whitcomb, M., Buchheit, B., Dorr, D., Malinoski, D., Korthuis, P. T., & Ono, S. S. (2023). Patient-Centered Telehealth Access to Opioid Use Disorder Health Care-a Qualitative Analysis. *Journal of General Internal Medicine*, *38*(Suppl 2), S318-S319. <https://dx.doi.org/10.1007/s11606-023-08226-z> | Abstract only (no full text published) |
| Lewer, D., Brothers, T. D., Croxford, S., Desai, M., Emanuel, E., Harris, M., & Hope, V. D. (2023). Opioid injection-associated bacterial infections in England, 2002–2021: a time series analysis of seasonal variation and the impact of coronavirus disease 2019. *Clinical Infectious Diseases*, *77*(3), 338-345. <https://doi.org/10.1093/cid/ciad144> | No intervention / insufficient details about intervention |
| Lodge, A., Partyka, C., & Surbey, K. (2022). A novel home-and community-based mobile outreach detoxification service for individuals identifying problematic substance use: implementation and program evaluation. *Canadian Journal of Public Health*, *113*(4), 562-568. <https://doi.org/10.17269%2Fs41997-022-00640-w> | Intervention not in response to COVID-19 pandemic |
| MacKenzie, O. W., Trimbur, M. C., & Vanjani, R. (2020). An isolation hotel for people experiencing homelessness. *New England Journal of Medicine*, *383*(6), e41. <https://doi.org/10.1056/NEJMc2022860> | Intervention not about substance use |
| Martin, A. K., Perryman, T., Bernstein, J. A., Taylor, J. L., Cruz, R., Muroff, J., Samet, J. H., & Assoumou, S. A. (2023). Peer recovery coaching for comprehensive HIV, hepatitis C, and opioid use disorder management: The CHORUS pilot study. *Drug and Alcohol Dependence Reports*, *7*, 100156. <https://doi.org/10.1016%2Fj.dadr.2023.100156> | Intervention not in response to COVID-19 pandemic |
| Martin, C., Andrés, P., Bullón, A., Villegas, J. L., de la Iglesia-Larrad, J. I., Bote, B., Prieto, N., & Roncero, C. (2021). COVID pandemic as an opportunity for improving mental health treatments of the homeless people. *International Journal of Social Psychiatry*, *67*(4), 335-343. <https://doi.org/10.1177/0020764020950770> | Intervention not about substance use |
| McDonald, R., Eide, D., Abel-Ollo, K., Barnsdale, L., Carter, B., Clausen, T., Day, E., Fonseca, F., Holmen, E., Horsburgh, K., Kelleher, M., Kåberg, M., Ladenhauf, M., McAuley, A., Metrebian, N., Neale, J., Parkin, S., Ratcliffe, K., Rintoul, C., Smith, J., … Strang, J. (2022). A rapid assessment of take-home naloxone provision during COVID-19 in Europe. *International Journal of Drug Policy*, *107*, e103787. <https://doi.org/10.1016/j.drugpo.2022.103787> | Doesn't meaningfully address the needs of people experiencing homelessness |
| Mejia-Lancheros, C., Lachaud, J., Gogosis, E., Thulien, N., Stergiopoulos, V., Da Silva, G., Nisenbaum, R., O’Campo, P., & Hwang, S. (2022). Providing housing first services for an underserved population during the early wave of the COVID-19 pandemic: a qualitative study. *PLoS One*, *17*(12), e0278459. <https://doi.org/10.1371/journal.pone.0278459> | No intervention / insufficient details about intervention |
| Mercer, T., Correa, M. F., Grove, L., Miller, J., Unroe, B., Kaiser, K., Benzer, J. K. (2022). Innovations in integrated care for individuals experiencing homelessness. *Journal of General Internal Medicine 37*(2), 577-578. <https://doi.org/10.1007/s11606-022-07653-8> | Intervention not in response to COVID-19 pandemic |
| Mericle, A. A., Sheridan, D., Howell, J., Braucht, G. S., Karriker-Jaffe, K., & Polcin, D. L. (2020). Sheltering in place and social distancing when the services provided are housing and social support: The COVID-19 health crisis and recovery housing. *Journal of Substance Abuse Treatment*, *119*, e108094. <https://doi.org/10.1016/j.jsat.2020.108094> | No intervention / insufficient details about intervention |
| Noel, M., Abbs, E., Suen, L., Samuel, L., Dobbins, S., Geier, M., & Soran, C. S. (2023). The Howard Street Method: A Community pharmacy-led low dose overlap buprenorphine initiation protocol for individuals using fentanyl. *Journal of Addiction Medicine*, *17*(4), e255-e261. <https://doi.org/10.1097/adm.0000000000001154> | Intervention not in response to COVID-19 pandemic |
| Novotna, G., Nielsen, E., & Berenyi, R. (2023). Harm reduction strategies for severe alcohol use disorder in the context of homelessness: A rapid review. *Substance Abuse: Research and Treatment*, *17*. <https://doi.org/10.1177%2F11782218231185214> | Review article |
| Noyes, E., Yeo, E., Yerton, M., Plakas, I., Keyes, S., Obando, A., Gaeta, J. M., Taveras, E. M., & Chatterjee, A. (2021). Harm reduction for adolescents and young adults during the COVID-19 pandemic: a case study of community care in reach. *Public Health Reports*, *136*(3), 301-308. <https://doi.org/10.1177/0033354921999396> | No intervention / insufficient details about intervention |
| Nygaard-Christensen, M., & Houborg, E. (2023). Pandemic lockdown as policy window for street-level innovation of health and substitution treatment services for people who use drugs. *Drugs, Habits and Social Policy*, *24*(3), 232-245. <https://doi.org/10.1108/DHS-03-2023-0008> | No intervention / insufficient details about intervention |
| Oreper, S., Bond, A., Bazinski, M., Tierney, M., Fang, M., Sankaran, S., & Rambachan, A. (2023). A Focused Screening and Clinical Intervention with Streamlined Outpatient Linkage for Hospitalized Patients with Opioid Use Disorder Experiencing Homelessness. *Substance Abuse: Research and Treatment*, *17*. <https://doi.org/10.1177%2F11782218231166382> | Intervention not in response to COVID-19 pandemic |
| Parkes, T., Carver, H., Masterton, W., Booth, H., Ball, L., Murdoch, H., Falzon, D., Pauly, B. M., & Matheson, C. (2021). Exploring the potential of implementing managed alcohol programmes to reduce risk of COVID-19 infection and transmission, and wider harms, for people experiencing alcohol dependency and homelessness in Scotland. *International Journal of Environmental Research and Public Health*, *18*(23), e12523. <https://doi.org/10.3390/ijerph182312523> | No intervention / insufficient details about intervention |
| Peavy, K. M., Darnton, J., Grekin, P., Russo, M., Green, C. J. B., Merrill, J. O., Fotinos, C., Woolworth, S., Soth, S., & Tsui, J. I. (2020). Rapid implementation of service delivery changes to mitigate COVID-19 and maintain access to methadone among persons with and at high-risk for HIV in an opioid treatment program. *AIDS and Behavior*, *24*(9), 2469-2472. <https://doi.org/10.1007/s10461-020-02887-1> | Doesn't meaningfully address the needs of people experiencing homelessness |
| Peterkin, A., Jawa, R, You, J. G., Cabral, H. J., Ruiz-Mercado, G., Park, T. W., Weinstein, Z. (2022). Pre-paid phone distribution to people with substance use disorder. *Journal of Addiction Medicine*, *16*(5), 304-305. <https://doi.org/10.1097/ADM.0000000000001068> | Updated version of the same article available for review |
| Polcin, D. L., Mahoney, E., Wittman, F., Sheridan, D., & Mericle, A. A. (2021). Understanding challenges for recovery homes during COVID-19. *International Journal of Drug Policy*, *93*, 102986. <https://doi.org/10.1016%2Fj.drugpo.2020.102986> | No intervention / insufficient details about intervention |
| Roncero, C., Vicente-Hernández, B., Casado-Espada, N. M., Aguilar, L., Gamonal-Limcaoco, S., Garzón, M. A., Martínez-González, F., Llanes-Álvarez, C., Martínez, R., & Franco-Martín, M. (2020). The impact of COVID-19 pandemic on the Castile and Leon addiction treatment network: a real-word experience. *Frontiers in Psychiatry*, *11*, e575755. <https://doi.org/10.3389/fpsyt.2020.575755> | Doesn't meaningfully address the needs of people experiencing homelessness |
| Rosmarin-DeStefano, C. L., Scarinci, E., Finkel, D., & Escabi, E. (2020). Risk Reduction service delivery during the COVID Pandemic to a marginalized urban population in Newark, NJ. *Open Forum Infectious Diseases*, *7*(1), 771. <https://doi.org/10.1093/ofid/ofaa439.1723> | Abstract only (no full text published) |
| Roxburgh, A., Jauncey, M., Day, C., Bartlett, M., Cogger, S., Dietze, P., Nielsen, S., Latimer, J., & Clark, N. (2021). Adapting harm reduction services during COVID-19: lessons from the supervised injecting facilities in Australia. *Harm Reduction Journal*, *18*, 20. <https://doi.org/10.1186/s12954-021-00471-x> | Intervention not about substance use |
| Rueda, Z. V., Haworth-Brockman, M., Sobie, C., Villacis, E., Larcombe, L., Maier, K., Deering, K., Sanguins, J., Templeton, K., & MacKenzie, L. (2023). Social and structural barriers and facilitators to HIV healthcare and harm reduction services for people experiencing syndemics in Manitoba: study protocol. *BMJ Open*, *13*(8), e067813. <https://doi.org/10.1136/bmjopen-2022-067813> | No intervention / insufficient details about intervention |
| Salisbury-Afshar, E. M., Rich, J. D., & Adashi, E. Y. (2020). Vulnerable populations: Weathering the pandemic storm. *American Journal of Preventive Medicine*, *58*(6), 892-894. <https://doi.org/10.1016/j.amepre.2020.04.002> | No intervention / insufficient details about intervention |
| Saloner, B., Krawczyk, N., Solomon, K., Allen, S. T., Morris, M., Haney, K., & Sherman, S. G. (2022). Experiences with substance use disorder treatment during the COVID-19 pandemic: Findings from a multistate survey. *International Journal of Drug Policy*, *101*, e103537. <https://doi.org/10.1016/j.drugpo.2021.103537> | No intervention / insufficient details about intervention |
| Saunders, E. C., Satcher, M. F., Monico, L. B., McDonald, R. D., Springer, S. A., Farabee, D., Gryczynski, J., Nyaku, A., Reeves, D., Kunkel, L. E., Schultheis, A. M., Schwartz, R. P., Lee, J. D., Marsch, L. A., & Waddell, E. N. (2022). The impact of COVID-19 on the treatment of opioid use disorder in carceral facilities: a cross-sectional study. *Health & Justice*, *10*(1), 35. <https://doi.org/10.1186/s40352-022-00199-1> | Doesn't meaningfully address the needs of people experiencing homelessness |
| Sellwood, T. (2022). Fast water and fast times in south East Queensland: Wrap around emergency toxicological, addiction and mental healthcare amid a pandemic and natural disaster. *Australian Nursing and Midwifery Journal*, *27*(8), 46. <https://search.informit.org/doi/10.3316/informit.520990841786848> | Doesn't meaningfully address the needs of people experiencing homelessness |
| Silliman Cohen, R. I., & Bosk, E. A. (2020). Vulnerable youth and the COVID-19 pandemic. *Pediatrics*, *146*(1), e20201306. <https://doi.org/10.1542/peds.2020-1306> | No intervention / insufficient details about intervention |
| Steer, K. J., Klassen, D. C., O’Gorman, C. M., Webster, M., Mitchell, M., Krichevsky, L., Christiansen, K., Benham, J. L., & Schindler, R. S. (2021). Cups for COVID: rapid implementation of a harm reduction initiative to support populations experiencing homelessness during the COVID-19 pandemic. *Canadian Journal of Public Health*, *112*(1), 29-35. <https://doi.org/10.17269/s41997-020-00466-4> | Intervention not about substance use |
| Stiles-Shields, C., Batts, K. R., Reyes, K. M., Archer, J., Crosby, S., Draxler, J. M., Lennan, N., & Held, P. (2022). Digital screening and automated resource identification system to address COVID-19–related behavioral health disparities: feasibility study. *JMIR Formative Research*, *6*(6), e38162. <https://doi.org/10.2196%2F38162> | Intervention not about substance use |
| Tanz, L. J., Jones, C. M., Davis, N. L., Compton, W. M., Baldwin, G. T., Han, B., & Volkow, N. D. (2023). Trends and characteristics of buprenorphine-involved overdose deaths prior to and during the COVID-19 pandemic. *JAMA Network Open*, *6*(1), e2251856. <https://doi.org/10.1001%2Fjamanetworkopen.2022.51856> | No intervention / insufficient details about intervention |
| Volkow, N., Weiss, S., Tapert, S., Chang, L., Walsh, S., D'Onofrio, G., Koustova, E., & Friedmann, P. (2020). COVID-19 and substance use disorders: intertwined epidemics. *Neuropsychopharmacology 45*(Suppl 1), 61. <https://doi.org/10.1038/s41386-020-00889-0> | Abstract only (no full text published) |
| Wiessing, L. G., Seguin-Devaux, C., & Merendeiro, C. S. (2021). Could the COVID-19 crisis help eradicate chronic homelessness? *American Journal of Public Health*, *111*(1), 25-26. <https://doi.org/10.2105%2FAJPH.2020.306015> | No intervention / insufficient details about intervention |
| Wilson, C. G., Ramage, M., & Fagan, E. B. (2021). A primary care response to COVID‐19 for patients with an opioid use disorder. *The Journal of Rural Health*, *37*(1), 169-171. <https://doi.org/10.1111/jrh.12438> | Doesn't meaningfully address the needs of people experiencing homelessness |
| Wise, J. M., Benning, L., Friedman, M. R., Wilson, T., Ramirez, C., Adimora, A., Stosor, V., Aouizerat, B., Sharma, A., Merenstein, D., Mimiaga, M., Sheth, A., Plankey, M., Cohen, M., Weiss, D. J., D’Souza, A., & Kempf, M. (2023). Disruptions in health care among MWCCS participants during the COVID-19 pandemic. *Conference on Retroviruses and Opportunistic Infection, 483,* 409. <https://www.croiconference.org/wp-content/uploads/sites/2/resources/2023/croi2023-abstract-ebook-v2.pdf> | No intervention / insufficient details about intervention |
| Wynn, Y., & Stergiopoulos, V. (2021). Optimizing care for people experiencing homelessness and serious mental illness amidst COVID-19: A street outreach perspective. *Journal of Health Care for the Poor and Underserved*, *32*(4), 1752-1763. <https://doi.org/10.1353/hpu.2021.0164> | No intervention / insufficient details about intervention |
| Zulman, D., Van Campen, J., Ferguson, J. M., Dhanani, Z., Greene, A. L., Kimerling, R., Slightam, C. (2023). Necessary but not sufficient: implementation and effectiveness of VA's digital divide initiative during the Covid-19 pandemic. *Journal of General Internal Medicine,* *38*(Suppl 2), 145. <https://doi.org/10.1007/s11606-023-08226-z> | Intervention not about substance use |
